# Long-term comparative analysis of AAV9-mediated gene replacement therapies for spinal muscular atrophy in mice

**DOI:** 10.1038/s41467-026-73545-8

**Published:** 2026-05-23

**Authors:** Xiupeng Chen, Qing Xie, Sarah J. Nath, Mojiao Tang, Hong Ma, Yasemin Özgür Günes, Tapan Sharma, Hao Liu, Mengtian Cui, Ailing Du, Mengjia Lu, Sophia Y. Liu, Boonying Wassamon, Mengyao Xu, Joseph Yunxi Wu, Qin Su, Timothy P. Fitzgibbons, Jinghua Liu, Fang Wan, Veena Kumanan, Ran He, Yijie Ma, Jun Yang, Heather L. Gray-Edwards, Thomas L. Gallagher, Phillip W. L. Tai, Guangping Gao, Jun Xie

**Affiliations:** 1 Horae Gene Therapy Center, Department of Genetic and Cellular Medicine, UMass Chan Medical School, Worcester, MA USA; 2 Li Weibo Institute for Rare Diseases Research, UMass Chan Medical School, Worcester, MA USA; 3 Viral Vector Core, UMass Chan Medical School, Worcester, MA USA; 4 Department of Microbiology, UMass Chan Medical School, Worcester, MA USA; 5 Division of Cardiovascular Medicine, Department of Medicine, UMass Chan Medical School, Worcester, MA USA; 6 CANbridge Pharmaceuticals, Burlington, MA USA

**Keywords:** Gene therapy, Motor neuron disease

## Abstract

Spinal muscular atrophy (SMA) results from a deficiency of the survival motor neuron (SMN) protein. Zolgensma, an adeno-associated virus (AAV)-based *SMN1* gene-replacement therapy, is approved for SMA, though its long-term efficacy and safety remain uncertain. This study compares a Zolgensma-like benchmark vector with a 2nd-generation vector featuring a codon-optimized *SMN1* transgene under the control of an endogenous *SMN1* promoter. In SMA mice, intracerebroventricular delivery of the 2nd-generation vector improved survival and phenotypic outcomes compared with the benchmark. However, motor impairment was observed in wild-type mice 20 months post-injection with the 2nd-generation vector. Notably, cardiac thrombosis and hepatocellular carcinoma were associated with the benchmark vector, but not with the 2nd-generation vector. While AAV-related tumorigenesis appears to be species-specific to mice, these findings underscore the need for careful long‑term monitoring in patients treated with Zolgensma.

## Abstract (web summary)

In mice, a next-generation gene therapy for spinal muscular atrophy improved survival and motor function more than a Zolgensma-like treatment, with fewer signs of liver and heart toxicity, supporting its promise as a safer, more effective approach.

## Introduction

Spinal muscular atrophy (SMA) is a hereditary neuromuscular disorder caused by biallelic loss-of-function of the Survival Motor Neuron 1 (*SMN1*) gene, resulting in insufficient survival motor neuron (SMN) protein. The disease primarily affects lower motor neurons in the anterior horn of the spinal cord and leads to progressive, symmetric muscle weakness and atrophy^1^. With an incidence of roughly 1 in 11,000 live births, SMA remains one of the most serious genetic causes of infant mortality in the absence of treatment^2–4^. Although motor neuron degeneration is central to disease pathogenesis, SMN is broadly expressed across tissues, and its physiological function extends beyond the central nervous system^4–13^. SMN is required for many fundamental cellular processes, including small nuclear ribonucleoprotein assembly, pre-mRNA splicing, axonal mRNA trafficking, and translational regulation^14–19^. Importantly, both insufficient and excessive SMN expression may be detrimental: while reduced SMN drives disease, overexpression has been associated with toxicities in the liver, heart, and dorsal root ganglion (DRG)^10,20–24^. Thus, these observations support the rationale for therapeutic strategies that restore SMN in a more regulated, near-physiological manner in clinically relevant tissues.

Onasemnogene abeparvovec (Zolgensma) is an intravenously administered AAV9-based gene replacement therapy approved for young children (<2 years old) with SMA carrying biallelic *SMN1* mutations^25–27^. Zolgensma is a self-complementary adeno-associated virus serotype 9 (scAAV9) vector that delivers the *SMN1* transgene under the control of a potent and ubiquitous cytomegalovirus enhancer/chicken β-actin (*CMVen*/*CB*) promoter. Zolgensma produced major clinical gains, including improved survival and attainment of motor milestones^28–30^. However, the therapeutic benefit of high-level systemic transgene delivery has been accompanied by important safety concerns. The current prescribing information includes a boxed warning for serious liver injury and acute liver failure, and liver-related adverse events have been commonly reported in clinical development^25–27^. In addition, signs of cardiac damage, indicated by an increased troponin I level in the serum, have been observed among treatment-emergent adverse events^20,31^. Notably, hepatic and cardiac toxicities were observed in preclinical studies, underscoring the need for next-generation SMA gene therapies that preserve efficacy while improving control over tissue exposure and expression levels^32^.

One reason Zolgensma is restricted to patients under 2 years old is that the dose is based on a patient’s weight. Older individuals require high doses of viral vectors, which would cause severe, potentially lethal toxicity. Injecting AAV vectors directly into the cerebrospinal fluid may achieve gene transfer throughout the CNS while reducing transduction in peripheral organs^33^. Itvisma^®^ is an intrathecal version of Zolgensma and was recently approved by the FDA for the treatment of SMA in patients aged 2–18 years^34^. However, the therapeutic benefits are moderate^35,36^. Administration of high doses of AAV through IV or intracerebroventricular (ICV) injection has been reported to cause neurodegeneration of the DRG in non-human primates (NHPs), indicating that DRG toxicity is a significant consideration for developing AAV vectors for clinical use^37–39^. ICV injection in mice showed that AAV9-mediated SMN overexpression achieved short-term success in ameliorating motor neuron degeneration but led to unexpected deterioration in motor behavior after 6 months^21^. This outcome is thought to mainly be caused by the loss of proprioceptive DRG neurons. AAV genomes persist primarily as episomes, with low-frequency integration (0.1–0.5%)^40,41^. AAV integration-related hepatocellular carcinomas (HCCs) have been observed in several murine studies^42^. However, HCC development has not been observed in other species such as dogs, NHPs, and human patients^43–45^. Thus, long-term animal studies are essential to assess the efficacy and safety of AAV-mediated SMA gene therapies.

We previously reported an AAV9 vector that expresses a codon-optimized *SMN1* transgene with a promoter derived from the *SMN1* gene as a 2nd-generation (2nd-G) gene therapy vector for SMA, which outperforms a vector similar to the design of Zolgensma (benchmark vector, BMK)^22^. The *SMN1* promoter enables *SMN1* transgene to express near-endogenous levels of SMN, which may be preferable to the ubiquitous promoter used in Zolgensma following IV administration. We previously reported a head-to-head comparison that showed that the 2nd-generation vector had a better safety profile (no increase in transaminase levels, a marker for liver damage) and improved efficacy (lower effective dose, broader therapeutic window, longer life span, minimal peripheral tissue pathologies, and increases in cardiac, respiratory, and motor functions) compared to the benchmark vector. Our data indicate that the 2nd-generation vector offers substantially improved therapeutic benefits over Zolgensma in a mouse model of SMA (SMNΔ7 mice)^22^.

Here, we extend our head-to-head comparison of the benchmark and 2nd generation vector by evaluating their long-term therapeutic efficacy and safety following ICV administration in SMA mice (12-month study) and in wild-type (WT) mice (20-month study). Our data indicate that: (1) the 2nd-generation vector outperformed the benchmark vector in SMA mice (*e.g*., longer lifespan, minimal peripheral tissue pathologies, and improved motor functions); (2) the 2nd-generation vector achieved more sustained SMN expression than the benchmark vector in both SMA and WT mice; (3) the benchmark vector induced cardiac thrombosis in both SMA and WT mice, but the 2nd-generation vector did not; (4) the 2nd-generation vector improved the motor function of SMA mice at 12 months post-treatment, but impaired WT mice at 20 months post-treatment; and (5) WT mice receiving the benchmark vector developed liver and lung tumors, but those receiving the 2nd-generation vector did not. However, the tumorigenesis caused by the benchmark vector may be a false alarm for Zolgensma, since AAV-integration-associated tumor risks have only been identified in murine models.

## Results

### The 2nd-G vector is more effective than the benchmark vector in SMA mice

**Fig. 1:**
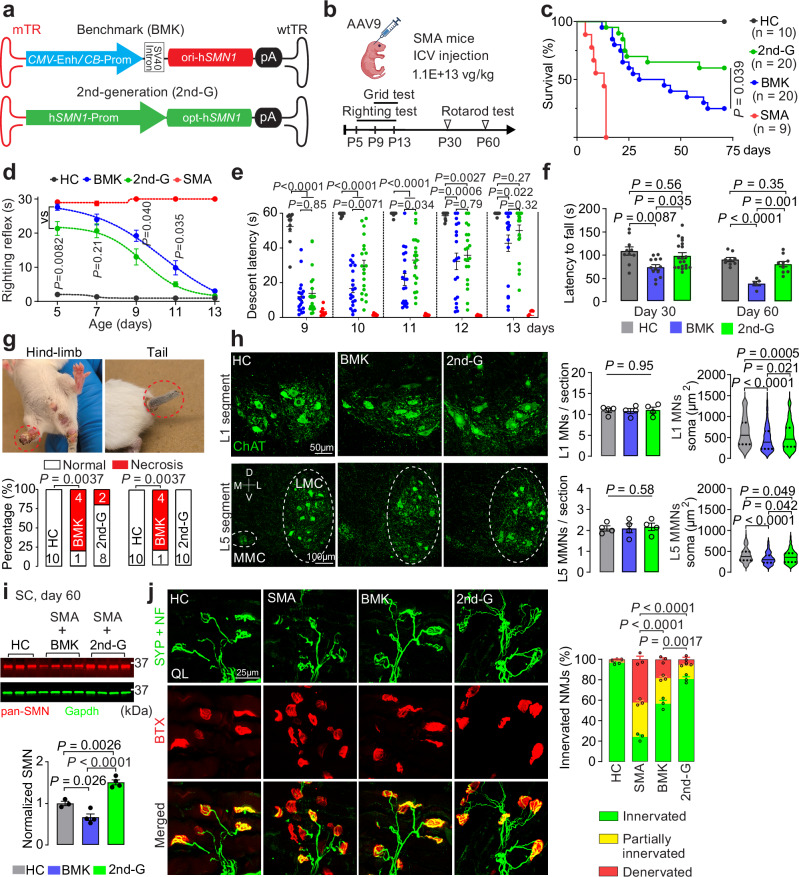
Therapeutic efficacy comparison in SMA mice. **a** Benchmark (BMK) and 2nd-generation (2nd-G) rAAV vectors. **b** Schematic of experimental design in the SMA (SMN∆7) mice. Mice were treated at P0 with 1.1E + 13 vg/kg by ICV injection. Non-treated SMA mice (SMA) and healthy carriers (HC) were used as controls. **c** Kaplan-Meier survival curves for the SMA mice receiving the BMK or 2nd-G vector in a 75-day study period, Log-rank (Mantel-Cox) test. **d**, **e** Surface righting reflex and grid test of treated mice (*n* = 10 in SMA, *n* = 10 in HC, *n* = 20 in BMK, *n* = 20 in 2nd-G). **f** Rotarod testing of the treated mice on days 30 and 60 (on day 30: *n* = 13 in BMK, *n* = 20 in 2nd-G, *n* = 10 in HC; on day 60: *n* = 5 in BMK, *n* = 10 in 2nd-G, *n* = 9 in HC). **g** Representative images of hind-limb and tail necrosis and frequencies of occurrence in each group. **h** Immunostaining with ChAT in the spinal cord (SC) on day 60. The L1 and L5 segments are shown in the top and bottom panels. Four mice per group were used for quantitative analysis. To calculate soma size, ~85 and 40 motor neurons (MN) were used for L1 MNs and L5 MMNs, respectively, in each mouse. LMC lateral motor column, MMC medial motor column. Medial, M; Lateral, L; Dorsal, D; Ventral, V. **i** Both human and mouse SMN protein (pan-SMN) levels in the SC lumbar segment on day 60, n = 3 in HC, *n* = 4 in BMK, and 2nd-G. **j** Representative images of NMJ immunostaining from QL muscles with NF, SYP, and BTX. Three animals per group were analyzed on day 11 post-ICV injection with 1.1E + 13 vg/kg. The *P* values present in (**d**) were obtained by a two-tailed unpaired Student’s *t*-test, two-sided Fisher’s exact test in (**g**), one-way ANOVA followed by Tukey’s multiple comparisons test in (**e**, **f**, **h**, **i**), two-way ANOVA with Tukey’s post hoc test in (**j**). Error bars presented as mean ± SEM. Graphics (**a**, **b**) were adapted from a previous publication^22^ and modified for use here under a Creative Commons 4.0 license (http://creativecommons.org/licenses/by/4.0/).

To compare the therapeutic efficacy between the 2nd-generation and benchmark vectors, we dosed the SMNΔ7 SMA mice with 1.1E + 13 vg/kg via ICV injection at postnatal day (P)0 and monitored their lifespan, motor function, and peripheral tissue pathologies (Fig. 1a, b). Non-treated diseased littermates (SMA) were used as controls. In an initial 75-day study period, 60% of the mice injected with the 2nd-generation vector survived (12 of 20), while only 25% in the benchmark-treated group (5 of 20) and 0% in the non-treated SMA group survived (Fig. 1c). Five days after injection, SMA mice that were treated with the 2nd-generation vector started to show the ability to right themselves, but the benchmark-treated group did not. Between 7–11 days post-injection, benchmark vector-treated SMA mice began to show improvements in the righting test, whereas the 2nd-generation vector-treated group consistently performed better. On day 13 post-injection, SMA mice from both treatments exhibited comparable performances on the righting test as the healthy carriers (HC) (Fig. 1d). Similar results were found for the surface grid test, as the 2nd-generation vector produced better results than the benchmark vector on days 10 and 11 post-injection, but comparable results on days 12 and 13. However, the 2nd-generation, but not the benchmark group performed comparable as the healthy carriers on day 13 (Fig. 1e). Latency to fall on the rotarod test was measured on days 30 and 60 post-injection, where the 2nd-generation vector-treated SMA mice performed similarly as their healthy carriers and outperformed the benchmark vector-treated group (Fig. 1f). In addition to improvements in lifespan and motor function with the 2nd-generation vector, we also observed less peripheral tissue damage, such as necrosis in the hind-limbs and tails. In the benchmark vector-treated group, 80% of the SMA mice developed hind-limb and tail necrosis. In contrast, 20% of the 2nd-generation vector-treated SMA mice exhibited hind-limb necrosis, but none showed signs of tail necrosis (Fig. 1g).

In human SMA patients, motor neuron (MN) degeneration is the hallmark; especially, MNs in the lumbar segment 1 (L1) and medial motor neurons (MMNs) in L5 are vulnerable, while L5 lateral motor neurons (LMNs) are more resistant^46–48^. Therefore, we examined them in the spinal cord (SC) 60 days after injection using Choline acetyltransferase (ChAT) immunostaining. We observed that both vectors restore the quantity of MN in L1, L5 MMNs, and L5 LMNs, but the 2nd-generation vector outperformed the BMK vector in rescuing soma size in both vulnerable and resistant motor neurons, approaching the levels observed in healthy carriers (Fig. 1h and Supplementary Fig. 1). Non-treated SMA mice did not survive long enough for comparisons on day 60. Western blot analysis of spinal cord tissue from the lumbar segment revealed that the benchmark vector produced less SMN protein than healthy carriers, whereas the 2nd-generation vector group produced more (Fig. 1i). We next examined the neuromuscular junctions (NMJs) of the quadratus lumborum (QL) muscles, which are known to be severely affected in SMA mice^49,50^. We assessed NMJ integrity in the QL muscles by staining the presynaptic NMJs with antibodies to synaptophysin (SYP) and neurofilament medium chain (NF-M), and the postsynaptic NMJs with α-bungarotoxin (α-BTX). On day 11 post-injection, both vectors can partially restore NMJ innervation in SMA mice, and the 2nd-generation vector was more effective (Fig. 1j). Together, these data indicate that the 2nd-generation vector shows greater therapeutic efficacy in extending survival, improving motor function, preventing peripheral tissue damage, and restoring NMJ morphology than the benchmark vector in SMA mice.

### The 2nd-G vector achieves sustained therapeutic outcomes in SMA mice

**Fig. 2:**
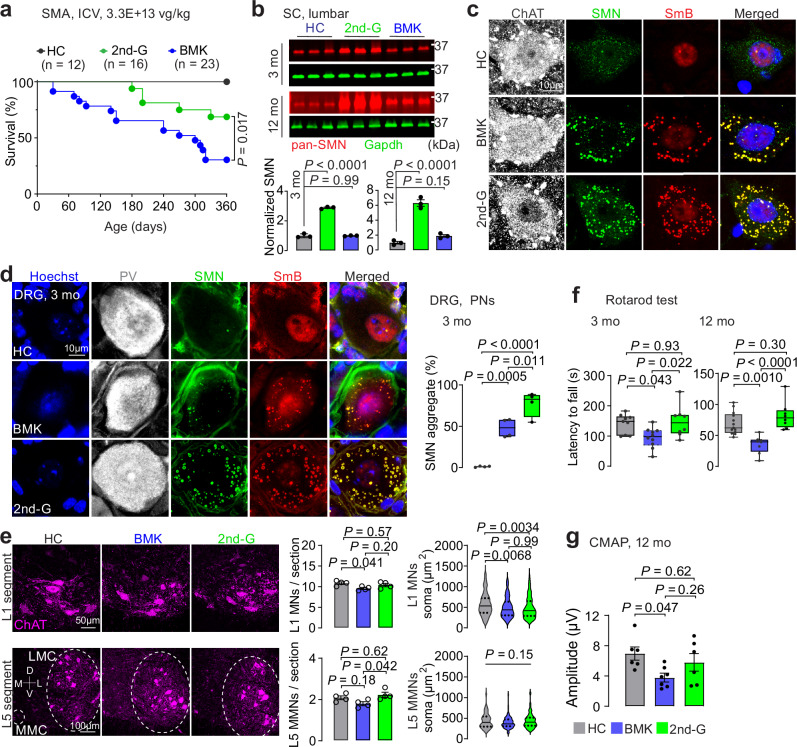
Comparison of long-term efficacy in SMA mice. **a** Kaplan-Meier survival curve of the SMA mice and HC in a 12-month study period. All SMA mice were treated at P0 at a dose of 3.3E + 13 vg/kg by ICV injection. **b** Western blot analysis of pan-SMN protein expression in the SC of SMA mice treated for three and 12 months, *n* = 3. **c** Immunostaining with SMN and SmB in ChAT^+^ motor neurons in the L5 lumbar spinal cord segment from the SMA mice post-injection for three months. **d** Immunostaining of SMN, PV and SmB in the L5 DRGs from the SMA mice post-injection for three months. Four mice in each group, PV^+^ neurons were used to quantify the SMN aggregates (*n* = 309 in HC, *n* = 244 in BMK, *n* = 279 in 2nd-G). **e** Immunostaining of MNs in the L1 and L5 segments from the SMA mice same as in (**a**). Four mice in each group. MN numbers in L1 MNs (*n* = 303 in HC, *n* = 267 in BMK, *n* = 290 in 2nd-G) and L5 MMNs (*n* = 58 in HC, *n* = 50 in BMK, n = 62 in 2nd-G). L1 MN soma size (*n* = 256 in HC, *n* = 189 in BMK, *n* = 224 in 2nd-G), L5 MMN soma size (*n* = 105 in HC, *n* = 81 in BMK, *n* = 129 in 2nd-G). The violin plots show the median (solid line) and interquartile range (dotted lines). **f** Rotarod test of the SMA mice three and 12 months after treatment. **g** Quantification of the compound muscle action potential (CMAP) from the mice same as in (**a**) (*n* = 6 in HC, *n* = 7 in BMK, *n* = 6 in 2nd-G). Log-rank (Mantel-Cox) test in (**a**) and one-way ANOVA followed by Tukey’s multiple comparisons test in (**b**, **d**–**g**). Box plots show the median (center line), interquartile range (box limits), and whiskers extending to the minimum and maximum values in (**d**, **f**). Bar plots are presented as mean ± SEM.

To compare the long-term therapeutic efficacy of the 2nd-generation and benchmark vectors, we increased the dose to 3.3E + 13 vg/kg and monitored SMA mice treated with these vectors for 12 months (mo). The median survival of benchmark vector-treated mice was ~300 days. In contrast, 68% of the 2nd-generation vector-treated SMA mice (11 of 16) survived for more than 360 days (Fig. 2a). Both vectors extended animal survival and improved SMA mice’s performance on righting and grid tests in a dose-dependent manner (Supplementary Fig. 2a–c). The higher-dose benchmark vector group showed better performance than the lower-dose group on the rotarod test. In contrast, the 2nd-generation vector groups exhibited comparable performance across both doses (Supplementary Fig. 2d). The 2nd-generation and benchmark vectors both achieved long-term SMN expression in the spinal cord at 90 and 360 days post-treatment. However, we detected more SMN protein expression in the 2nd-generation group (Fig. 2b). Compared to the benchmark-treated group, the 2nd-generation-treated group showed more SMN expression in the liver and brain, similar levels in the heart, and less expression in the quadriceps at post-injection day 40 and day 90 (Supplementary Fig. 3). AAV9-mediated overexpression of SMN by the ubiquitous β-glucuronidase (*GUSB*) promoter has been reported to cause hind-limb clasping, a neurological phenotype that results from the loss of proprioceptive synapses and neurodegeneration^21^. We therefore examined SMN expression in ChAT^+^ motor neurons and Parvalbumin-positive (PV+) proprioceptive neurons (PNs) in the L5 spinal segment and DRGs, respectively. By co-staining with SMN, SmB (a core member of the small nuclear ribonucleoproteins that form the SmB/SMN complex)^51,52^, we observed “Gemini of Cajal bodies (gems)-like” puncta of SMN in both the cytoplasm and nucleus of ChAT^+^ and PV^+^ neurons that received either vector for 90 days, but more SMN cytoplasmic aggregates were found in the 2nd-generation-treated group compared to the benchmark-treated group after three months of treatment (Fig. 2c, d). We detected SMN aggregates in 47% and 76% of PV^+^ neurons in the benchmark and 2nd-generation vector groups, respectively (Fig. 2d). Western blot analysis confirmed more SMN expression in DRGs from the 2nd-generation vector (Supplementary Fig. 4). After 12 months of treatment, we analyzed the quantity and soma sizes of MN in the L1 spinal cord, L5 MMNs, and L5 LMNs. Compared with the healthy carriers, the 2nd-generation vector group exhibited comparable cell numbers of L1 MNs, L5 MMNs, and L5 LMNs, whereas the benchmark vector group had fewer L1 MNs. Compared to the 2nd-generation vector group, the benchmark vector treatment showed comparable L1 MNs and L5 LMNs, but fewer L5 MMNs. The soma size of L1 MNs in both vector-treated mice is smaller than that of healthy controls, but their soma size of L5 MMNs and L5 LMNs is comparable to that of the healthy controls (Fig. 2e and Supplementary Fig. 5). Immunostaining with ChAT^+^ and vesicular glutamate transporter 1 (VGluT1), which is a marker in various neurodegenerative disease^53^, showed no apparent differences between the AAV-treated SMA mice and healthy carriers (Supplementary Fig. 6). No occurrence of hind-limb clasping in the treated SMA mice was found after 12 months (Supplementary Fig. 7). Importantly, SMA mice that were treated with the 2nd-generation vector showed improved performance in the rotarod test compared to the benchmark group, achieving similar performances as the healthy controls in this 12-month study period (Fig. 2f). The motor function improvement was supported by electrophysiological evidence, which compound muscle action potential (CMAP) amplitude from the 2nd-generation vector treatment is comparable with the healthy controls, but not the benchmark group (Fig. 2g). We further increased the dose to 1.0E + 14 vg/kg and treated the SMA mice through ICV with at P0. In a 90-day study, all animals (*n* = 7) survived in the 2nd-generation vector group, and one mouse (*n* = 7) died in the benchmark group (Supplementary Fig. 8a). No differences in body weight gain or the rotarod test were observed between these two groups. However, the 2nd-generation vector-treated mice, but not the benchmark treatment, exhibited performance on the rotarod test comparable to WT mice on day 90 (Supplementary Fig. 8b, c). Together, these data show that SMN overexpression is therapeutic and tolerated in SMA mice, with the 2nd-generation vector producing better outcomes than the benchmark vector.

### Supraphysiological SMN protein induces cardiac thrombosis

**Fig. 3:**
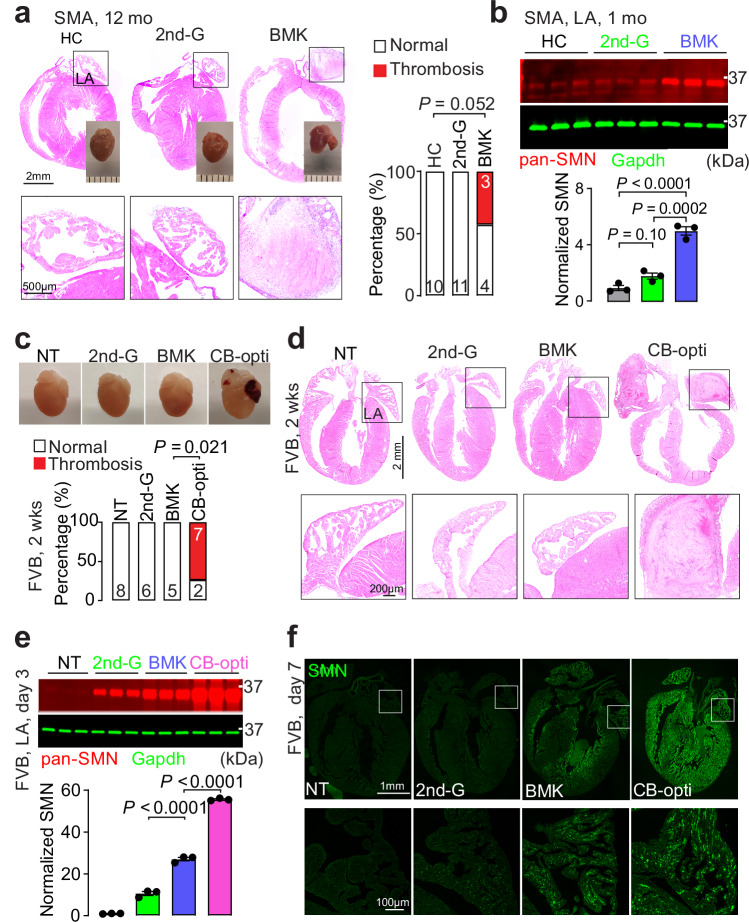
Supraphysiological SMN protein induces cardiac thrombosis in both SMA and WT mice. **a** H&E staining of mouse hearts from SMA mice treated with the benchmark vector at 3.3E + 13 vg/kg for 12 months. Occurrences by percentage are shown at the right. **b** SMN protein expression in the left atrium of SMA mice treated for one month by Western blot, three animals in each group. **c** Gross image of heart from FVB WT mice two weeks post-injection of a 1.0E + 14 vg/kg dose delivered via ICV administration at P0-P1. The frequency of left atrial thrombosis (LAT) is shown at the bottom. The CB-opti construct is a re-engineered version of the benchmark vector, created by replacing the native *SMN1* coding sequence with codon-optimized h*SMN1* to maximize SMN expression. NT, non-treated FVB mice. **d** H&E staining of the FVB mouse hearts two weeks after treatment. **e** SMN protein expression in the left atrium of FVB WT mice after receiving the AAV treatment for three days by ICV injection at 1.0E + 14 vg/kg at P0. Three animals in each group. **f** Immunostaining of the whole heart with anti-SMN antibody from FVB WT mice on day 7 post-injection. Bar plots in (**b**, **e**) represent mean ± SEM, and the statistical tests used were one-way ANOVA followed by Tukey’s multiple comparisons test. Two-sided Fisher’s exact test was used in (**a**, **c**).

We previously assessed heart function in SMA mice using echocardiography following IV-administered gene therapy^22^. We found that mice treated with the benchmark vector showed compromised cardiac function compared to healthy carriers. In contrast, mice treated with the 2nd-generation vector showed no abnormalities in heart function. The abnormality observed in the benchmark vector-treated group may result from supraphysiological cardiac SMN expression (>68-fold higher than in healthy controls after one month of treatment) following IV delivery^22^. After switching to ICV administration, cardiac SMN expression in benchmark-treated mice dramatically reduced to about one-fold higher than that of the healthy controls (Supplementary Fig. 3a). The ICV approach produced no premature ventricular contraction in the benchmark-treated group at 90- and 300-days post-treatment (Supplementary Fig. 9a). The ejection fraction and stroke volume in SMA mice treated with both vectors were comparable to those of healthy controls (Supplementary Fig. 9b). Surprisingly, we observed thrombosis in the left atrium in three out of seven surviving SMA mice (12 total) in the benchmark vector-treated group, but none of the 11 surviving SMA mice (16 total) in the 2nd-generation vector group showed thrombosis at 360 days post-treatment (Fig. 3a). We detected higher levels of SMN protein in the left atrium of SMA mice treated with the benchmark vector compared with those receiving the second-generation vector and with healthy controls (Fig. 3b). We therefore reasoned that long-term supraphysiological expression of SMN may be detrimental to cardiac function. To further assess the importance of maintaining physiological expression, we replaced the native coding sequence of *SMN1* in the benchmark vector genome with a codon-optimized cDNA to maximize SMN expression (CB-opti), as we had previously^22^. We increased the dose to 1.0E + 14 vg/kg for these three vectors and treated WT FVB mice by ICV injection at P0. After 2 weeks, 7 of 9 mice treated with the CB-opti vector developed left atrial thrombosis, whereas none of the mice receiving the benchmark or 2nd-generation vector did (Fig. 3c, d). We detected the highest SMN expression in the left atria of mice that received the CB-opti vector by Western blot analysis and immunostaining on days 3 and 7, respectively (Fig. 3e, f). Collectively, our data suggest that supraphysiological SMN expression is detrimental to cardiac function, leading to impaired cardiac function or atrial thrombosis.

### The 2nd-G vector impaired motor functions in WT mice

**Fig. 4:**
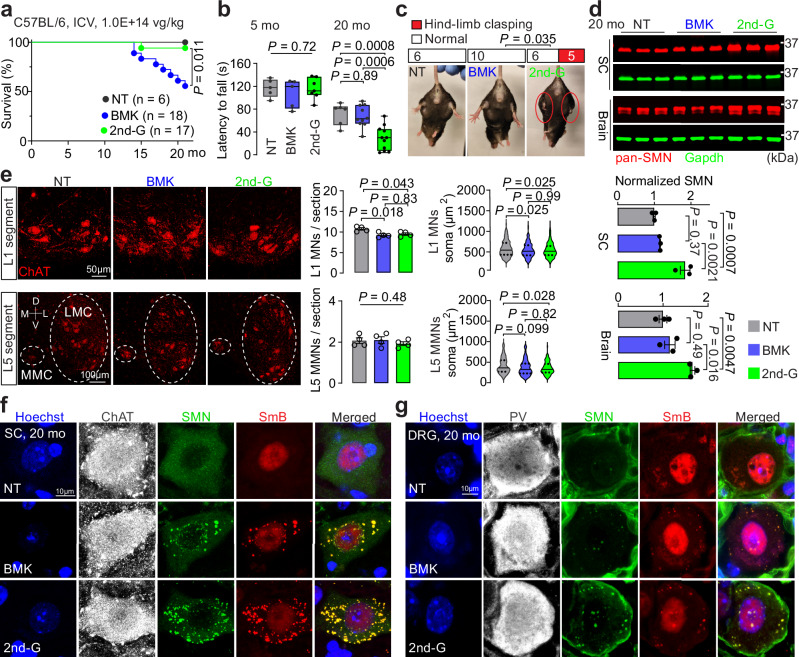
The 2nd-G vector impaired the motor function of WT mice. **a** Kaplan-Meier survival curve of C57BL/6 mice in a 20-month study period. All C57BL/6 mice were treated at P0 with 1.0E + 14 vg/kg via ICV injection. Non-treated (NT) healthy littermates were used for comparison. **b** Rotarod test at five and twenty months after treatment. Animals in 5 months: *n* = 5 in NT and BMK, *n* = 7 in 2nd-G. In 20 months: *n* = 6 in NT, n = 10 in BMK, *n* = 11 in 2nd-G. **c** Hind-limb clasping phenotype observed in C57BL/6 mice in 2nd-G vector-treated mice. **d** SMN protein expression in the spinal cord (SC) and brain of C57BL/6 mice after receiving the AAV treatment for 20 months. Quantification of SMN is shown at the bottom. Three animals in each group. **e** Immunostaining of ChAT^+^ motor neurons in the L1 and L5 segments from the C57BL/6 mice post-injection for twenty months. Four animals were included in each group in comparison. MN numbers were quantified in L1 MNs (*n* = 299 in NT, *n* = 257 in BMK, n = 264 in 2nd-G) and L5 MMNs (*n* = 58 in NT, *n* = 59 in BMK, *n* = 54 in 2nd-G). L1 MN soma size (*n* = 407 in NT, *n* = 290 in BMK, n = 311 in 2nd-G), L5 MMN soma size (*n* = 97 in NT, *n* = 124 in BMK, *n* = 110 in 2nd-G). **f** ChAT, SMN and SmB immunostaining of L5 spinal cord from C57BL/6 mice after receiving the AAV treatment for 20 months. **g** PV, SMN, and SmB immunostaining of L5 DRGs of C57BL/6 mice after receiving the AAV treatment for 20 months. Hoechst applied for nuclear counterstaining. Two-sided Fisher’s exact test in (**c**), bar plots are presented as mean ± SEM, and Box-and-whiskers graphs show the median, interquartile range, and minimum and maximum values. Log-rank (Mantel-Cox) test applied in (**a**), one-way ANOVA followed by Tukey’s multiple comparisons test in (**b**, **d**, **e**), and two-sided Fisher’s exact test was used in (**c**).

To further assess the long-term safety of AAV-mediated SMN expression, we injected the benchmark or 2nd-generation vector into neonatal C57BL/6 WT (P0) mice by ICV at a dose of 1.0E + 14 vg/kg and monitored the animals for 20 months. The dose of 1.0E + 14 vg/kg was chosen because the same dose of AAV9 vector expressing SMN resulted in neurological defects in WT mice in a long-term study^21^. WT mice without treatment (NT) were used as controls. During this period, 44% of mice in the benchmark group (8 of 18) and 6% of mice in the 2nd-generation group (1 of 17) died (Fig. 4a). No differences were observed in the rotarod test for either group compared with WT mice at 5 months after injection, but the group injected with the 2nd-generation vector showed a decline in performance at 20 months compared to the other two groups (Fig. 4b). Moreover, we observed that 2nd-generation vector injected mice presented hind-limb clasping phenotype, but none of them in the untreated or benchmark vector group (Fig. 4c). At 20 months post-injection, overexpression of SMN protein was detected in the brain and spinal cord of mice that received the 2nd-generation vector, but not in mice treated with the benchmark vector (Fig. 4d). In contrast, the transduced AAV vector genome copy numbers were found to be comparable in the spinal cords of both groups, indicating that the 2nd-generation vector genome design resulted in sustained SMN expression and the benchmark vector genome is inactive (Supplementary Fig. 10). We next examined the motor neurons in the spinal cord by ChAT immunostaining. Both vectors reduced the quantity of L1 MNs, but not L5 MMNs or L5 LMNs. Both vectors decreased the soma size of L1 MNs, but only the 2nd-generation vector reduced the soma size of L5 MMNs and L5 LMNs (Fig. 4e and Supplementary Fig. 11). Compared with WT controls, both vectors induced SMN/SmB cytoplasmic aggregates in ChAT^+^ and PV^+^ neurons, and more were observed in the 2nd-generation vector group (Fig. 4f, g). In the L5 lumbar segment, SMN aggregates are present in ~9% and ~62% of PV^+^ proprioceptive neurons in the benchmark and 2nd-generation group, respectively (Supplementary Fig. 12). In line with previously reported results^21^, our data provide evidence that long-term overexpression of SMN is associated with neurodegeneration and motor function impairment.

### The benchmark, but not the 2nd-G vector, induces hepatocellular carcinoma in WT mice

**Fig. 5:**
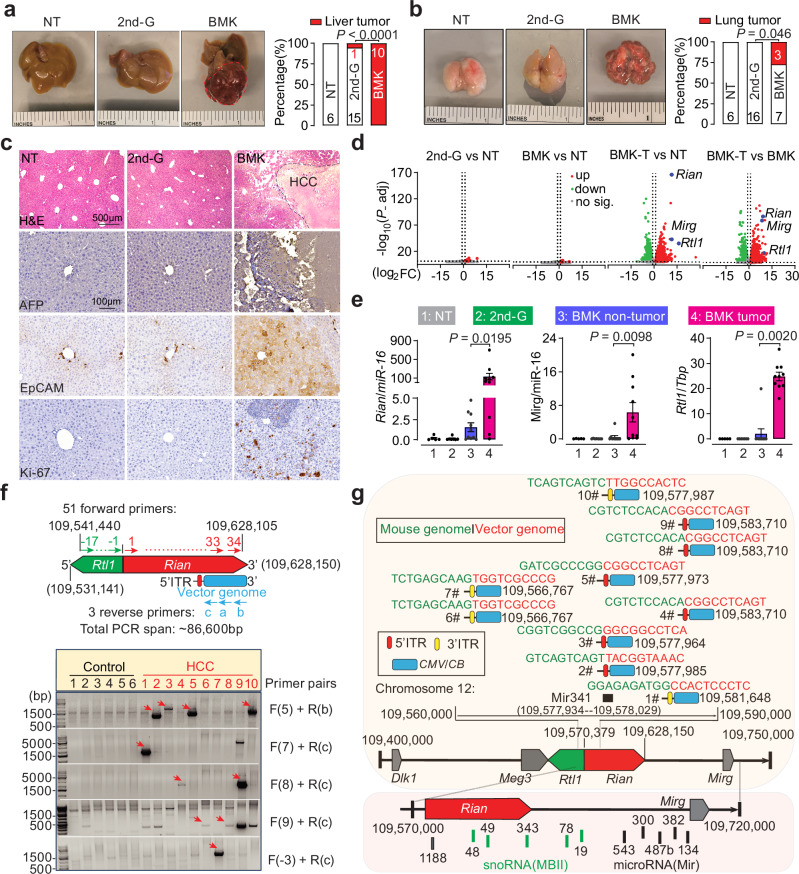
Benchmark vector integrates into *Rtl1-Rian* locus and induces hepatocellular carcinoma. **a, b** Gross images of the liver and lung from the C57BL/6 mice 20 months after receiving AAV treatment. All mice were treated at P0 by ICV injection at a dose of 1.0E + 14 vg/kg. Non-treated mice were used as controls. The percentage of animals with tumors is shown on the right. **c** H&E staining and immunohistochemistry (IHC) staining with Alpha Fetoprotein (AFP), epithelial cellular adhesion molecule (EpCAM), and Kiel 67 antibodies in the liver of mice that received AAV treatment for 20 months. Four mice per group were analyzed, and a representative image is shown. **d** Transcriptome comparison between the liver of the 2nd-G vector group vs. non-treated C57BL/6 mice, the BMK vector group vs non-treated C57BL/6 mice, and the liver tumor from the BMK vector group versus its adjacent liver or the non-treated group. FC, Fold Change, adjusted *P* values (*P*_adj) were calculated using the Benjamini–Hochberg (BH) method, and *P*_adj <0.05 was considered statistically significant, indicated by dashed lines in the graphs. **e** *Rian*, *Mirg*, and *Rtl1* expression by qRT-ddPCR in the liver and liver tumor tissues, *n* = 5 in NT, *n* = 7 in 2nd-G, *n* = 10 in BMK non-tumor, and in BMK tumor. Wilcoxon matched-pairs signed-rank test applied for analysis. **f** AAV genome integration analysis in the liver and liver tumor tissues from C57BL/6 mice treated with AAV vectors for 20 months. Primer design (top) and PCR amplicons (bottom) were used for Sanger sequencing, bp for base pair. **g** Vector-chromosome junctions and AAV genome integration sites in the mouse genome. Sequences in green and red are from the mouse genome and the vector genome, respectively. Two-sided Fisher’s exact test was used in (**a**, **b**).

We previously showed that IV delivery of the benchmark vector led to liver toxicity in mice because of the supraphysiological expression of SMN protein, while the 2nd-generation vector did not^22^. After changing the administration route from IV to ICV, no elevation in alanine transaminase (ALT) liver enzyme or liver pathology was detected in either of the benchmark or 2nd-generation vector treated SMA mice within the first three months after dosing (Supplementary Fig. 13a, b), which may be due to reduced hepatic SMN expression (Supplementary Fig. 3). However, after 20 months (Fig. 4a), all of the surviving animals (10 of 10) in the benchmark group developed liver tumors (Fig. 5a). In contrast, only one mouse in the 2nd-generation vector group (1 of 16) developed liver tumor. It is important to note that a low incidence of liver tumor (1–4%) is known to occur in older C57BL/6 mice^54^. Additionally, tumors were found in the lungs (3 of 10) (Fig. 5b). Significant elevation of ALT and aspartate aminotransferase (AST) was found in the benchmark vector-treated mice (AST: 620.2 ± 410.7 U/L and ALT: 1518.2 ± 1097.0 U/L). In contrast, no elevation was found in the 2nd-generation vector-treated mice (AST: 20.6 ± 3.0 U/L and ALT: 49.4 ± 6.1 U/L, *P* < 0.05 for both comparisons) (Supplementary Fig. 13c). Alpha-fetoprotein (AFP), which is a serum marker for liver cancer, was elevated in mice treated with the benchmark vector, but not in the other groups (Supplementary Fig. 13d). Histological analysis revealed that the tumors were hepatocellular carcinoma (HCC) containing abnormal hepatocytes and morphology (Fig. 5c). In addition to AFP, we also examined mouse liver using immunohistochemistry (IHC) for epithelial cellular adhesion molecule (EpCAM), which is a characteristic of some types of HCC^55^ and Ki-67, a cellular marker of proliferation. We observed that more hepatocytes were positive for AFP, EpCAM, and Ki-67 in the livers of mice treated with the benchmark vector than in those treated with the 2nd-generation vector or in non-treated mice (Fig. 5c). These results indicate that the benchmark vector caused liver tumors, whereas the 2nd-generation vector did not.

Long-term overexpression of SMN or integration of AAV vector DNA into the host genome may lead to HCC formation. We compared SMN expression in the livers of mice treated with either benchmark or 2nd-generation vector and non-treated controls 20 months after injection by western blot, and no differences were found between the three groups (Supplementary Fig. 13e). However, transcriptome analysis revealed increased expression of *Rian*, *Mirg*, and *Rtl1* in the liver tumors from benchmark vector-treated mice compared to the tumor-adjacent tissues and livers from non-treated WT mice (Fig. 5d). AAV genome integration into the mouse *Rian* locus has been proposed as a mechanism for carcinogenesis^41,42,56,57^. This locus encodes numerous regulatory RNAs, including microRNAs (miRNAs), small nucleolar RNAs (snoRNAs), and long non-coding RNAs (lncRNAs)^58^. Elevations in these genes are associated with hepatic genotoxicity related to AAV genome integration. In the benchmark-treated mouse liver tumors, qRT-PCR showed upregulation of *Rian*, *Mirg*, and *Rtl1*, suggesting that they were caused by AAV genome integrations in the *Rian* locus (Fig. 5e). We then analyzed the integration of AAV vector genomes in this part of the mouse genome (Fig. 5f). We designed primers that span the *Rtl1-Rian* region using 51 forward primers targeting the mouse genome and three reverse primers recognizing the benchmark vector genome. Analysis by PCR detected 10 integration events in tumor tissues, but none in tumor-adjacent tissues (indicated by red arrows in Fig. 5f). Sanger sequencing revealed the presence of inverted repeats (ITRs) in all of the integrated genomes, six containing the 5′-ITR and four containing the 3′-ITR (Fig. 5g). Of note, the same insertion site was detected in three mice (#4, #8, and #9). Another identical integration site was found in two other mice (#6 and #7) (Fig. 5g and Supplementary Fig. 14a), indicating the presence of integration hotspots in the *Rian* locus. Our qRT-PCR analysis demonstrated that delta-like non-canonical Notch ligand 1 (*Dlk1*), multiple miRNAs (miR-1188, miR-134, miR-543, miR-300, miR-487b, miR-382), and snoRNAs (MBII-19, MBII-49, MBII-343, MBII-78, MBII-48) were elevated in HCCs (Supplementary Fig. 14b). Taken together, our data indicate that ICV administration of the benchmark vector at P0 leads to HCC in mice due to vector genome integrations, whereas no HCC is observed in mice treated with the second-generation vector.

## Discussion

Efficacy, durability, and safety are critical factors in the development of AAV-mediated gene therapies for SMA. Here, we evaluate long-term therapeutic considerations for a benchmark vector, similar to Zolgensma (a human *SMN1* transgene with the native cDNA sequence driven by a strong constitutive promoter), and our 2nd-generation vector (a codon-optimized *SMN1* sequence driven by the human *SMN1* promoter). Both constructs were packaged into AAV9 and delivered via ICV to evaluate long-term therapeutic efficacy and safety in SMA (12-month study) and WT (20-month study) mice, respectively. Compared with the benchmark vector, the 2nd-generation vector showed improved efficacy (e.g., longer lifespan and enhanced motor function), more durable SMN expression, minimal peripheral tissue pathology, and no adverse effects on the heart and liver. However, the 2nd-generation vector led to motor function impairment in WT mice 20 months after treatment.

Zolgensma has demonstrated substantial clinical benefits, with safety concerns including liver, heart, and CNS toxicity in animal models and human patients^10,20,21,59^. One main mechanism underlying these adverse effects is immune responses to the high-dose AAV9 capsid, which are required for CNS. In addition, our previous study^22^ and another recent study^60^ suggest that excessive SMN expression from the Zolgensma-like vector leads to hepatic and cardiac toxicity in mice. These toxicities were resolved by using either the endogenous *SMN1* promoter or a neuron-specific promoter with cytomegalovirus (*CMV*) enhancer to reduce SMN expression in peripheral tissues^22,60^. Administration of Zolgensma-like vector into NHPs results in transient acute liver injury, but empty AAV9 or AAV9 with a promoter-less genome causes no transaminase elevation or microscopic changes. At the same time, immune suppression with prednisolone or rituximab/everolimus failed to block the hepatotoxicity of the Zolgensma-like vector^39^. This emerging evidence suggests that the controlled level of SMN expression is desired.

To cross the blood-brain barrier, very high doses of AAV9 must be administered intravenously. The majority of AAV9 genomes, upon IV injection, targeted the liver and heart, resulting in high vector load and transgene expression. In contrast, ICV injection is more effective at transducing the CNS, with substantially lower transduction in peripheral tissues, at a substantially lower dose than the IV route^61^. Changing the route of administration from IV to ICV mitigated the acute liver damage and heart function impairments caused by the benchmark vector in the SMA mice (Supplementary Fig. 13a, b, and Supplementary Fig. 9). However, we found that benchmark vector-treated SMA mice developed cardiac thrombosis 12 months after injection (Fig. 3a). This outcome was associated with supraphysiological expression of SMN in the heart (Fig. 3b–f), suggesting that overactivity of the protein and/or the stress of increased protein synthesis may be pathogenic. Similarly, we observed that the 2nd-generation vector led to overexpression of SMN in the CNS, which corresponded with the formation of SMN aggregates in motor and DRG PV^+^ neurons in both SMA and WT mice (Fig. 2c, d; Fig. 4f, g). At the end of the 12-month study, no SMA mice treated with either vector exhibited hind-limb clasping behavior, a neurological symptom attributed to DRG toxicity and severe neurodegeneration^21,62^. Importantly, 2nd-generation vector-treated SMA mice outperformed the benchmark vector-treated group and performed similarly to WT mice in the rotarod test 12 months after treatment (Fig. 2f). In contrast, WT mice given the 2nd-generation vector showed a notable decline in performance on the rotarod test and hind-limb clasping phenotype after treatment, but those given the benchmark vector did not (Fig. 4b, c). Although neurological toxicity was not observed in the SMA mice treated with the 2nd-generation vector, the motor function decline and hind-limb clasping in WT mice suggest that excessive SMN expression in the CNS and DRGs could be a concern for long-term health. High levels of human SMN overexpression from a high vector dose (1.0E + 14 vg/kg by ICV), combined with endogenous mouse Smn, may impair motor function, as adverse effects were observed only in WT mice at high dose.

The 2nd-generation vector may offer improved therapeutic benefits than the benchmark vector for SMA gene therapy by dynamically regulating SMN expression across different tissues. To further enhance AAV gene therapy for treating SMA, an AAV capsid with improved CNS tropism and the ability to de-target the DRG, such as the recently developed BI-hTFR1 capsid, may substantially improve efficacy and safety by reducing the dose required to achieve therapeutic efficacy^63^. The endogenous *SMN1* promoter enables the AAV-delivered *SMN1* gene to be expressed at levels closer to physiological, but the high tropism of AAV in certain tissues and cell types may result in multiple copies being expressed, leading to supraphysiological SMN levels and subsequent adverse effects. In our study, the 2nd-generation vector mitigates liver and heart toxicities observed with the benchmark vector; however, DRG toxicities may persist. The potential neurological toxicity of the 2nd-generation vector warrants further investigations on other species, such as rats, piglets, and NHPs, before clinical evaluation. To minimize DRG toxicity, DRG-specific de-targeting strategies, such as miR-183 binding sites, can be incorporated into the vector genome. This approach has been applied to eliminate DRG toxicity in NHPs without compromising therapeutic gene expression in the CNS^64^. In addition to the DRG, we observed that SMN aggregates in motor neurons following delivery of the 2nd-generation vector (Figs. 2, 4). To overcome deleterious effects caused by overexpression of SMN, incorporation of negative regulatory elements in the vector genome that can sense supraphysiological SMN expression or cellular stress may improve the safety^65^. To control the SMN expression after gene therapy delivery, drug-induced splicing^66^ or RNA switches^67–69^ can be incorporated into the therapeutic cargo. These considerations apply to gene replacement strategies but would be circumvented by gene-editing strategies^70,71^. Treatment strategies other than gene replacement: integration of the *SMN1* coding sequence into the host cell gene locus downstream of its endogenous promoter; restoration of SMN expression by editing the splicing of *SMN2*, which is a paralog of *SMN1*, to boost the endogenous SMN expression; and directly correcting the disease-causing *SMN1* mutation by gene editing.

In addition to safety and efficacy concerns arising from SMN misexpression in the CNS and peripheral tissues, we also observed a high incidence of HCC in animals treated with the benchmark vector. AAV-vector DNA integration-induced tumorigenesis appears to be species-specific because it occurs near the mouse *Rian* locus. Integration of *CMVen*/*CB* promoter into the *Rian* locus induced HCC in mice^56^; however, the *Rian* locus is absent from the genomes of other species. The integration sites of AAV in humans are random, with no preferential sites or regions associated with HCC^43,72,73^. The general risk of tumorigenesis in human patients and in specific disease states remains largely unknown, but several observations have been reported: chromosomal integrations of rAAV were found to be 1–3% in human hepatocytes, a surprisingly high frequency^74^. In this study, the AAV integration frequency was assessed in a humanized liver mouse model using a PCR-free, capture-sequencing approach coupled with long-read PacBio SMRT (single-molecule, real-time) sequencing. Compared to this innovative approach, the conventional PCR-based, short-read Illumina sequencing approach may underestimate the AAV integration frequency; a 16-month-old SMA child developed epithelioid neoplasm of the spinal cord with detectable vector genomes in the tumor tissues, but not all the tumor cells, approximately 14 months after receiving Zolgensma^75^; and a SMA patient was diagnosed with pilocytic astrocytoma at 2 years of age, approximately 8 months after Zolgensma treatment, but molecular analysis of the tumor tissue suggests no linkage between tumorigenesis and administration of Zolgensma^76^. Genotoxicity has been reported to be affected by rAAV vector design, including the selection of enhancer/promoter elements^41,77–79^, and the *CMVen*/*CB* promoter in the benchmark vector has been shown to cause hepatic genotoxicity in mice^41,77,79^. AAV vector integration with the *CMVen*/*CB* promoter alters the expression levels of several miRNAs encoded in the *Rian* locus and adjacent genes, leading to the development of HCC^41,56,79^. In line with previous studies^41^, our data suggest that careful selection of an appropriate promoter can mitigate AAV genome-integration-associated tumorigenesis. The high occurrence of HCC in the mice receiving the benchmark vector warrants long-term monitoring of SMA patients treated with Zolgensma.

The development of therapeutic approaches to treat SMA has benefited from the translatability of the SMA mouse models^25,80–83^. Evaluation of their long-term therapeutic efficacy and safety in pre-clinical studies remains critical and challenging, especially for Zolgensma and other AAV approaches, which are regarded as “one-and-done” treatments. Clinical data suggest that the therapeutic benefits of Zolgensma are limited to certain years^84^. In WT mice, we detected sustained SMN expression in the spinal cord after 20-month treatment with the 2nd-generation vector but not the benchmark vector (Fig. 4d), despite comparable transduced vector genomes (Supplementary Fig. 10). Epigenetic silencing of the vector genome could be the cause of loss of SMN expression from the benchmark vector^85,86^. *CMV* promoter is vulnerable to epigenetic silencing^87,88^. The *CMVen*/*CB* promoter in the benchmark vector genome may potentially loss its activity over time. Whether the 2nd-generation vector will provide more sustainable SMN expression needs to be further investigated. The tumorigenesis observed in the benchmark vector is concerning, but such events have not been reported in 10-year-long-term studies in dogs treated with AAV for hemophilia^44,45^. At present, AAV-induced tumorigenesis remains a theoretical risk in humans^89^. Intrathecal Zolgensma demonstrated improvements in motor function in SMA patients aged 2 to 18 years, with an acceptable safety profile^35,36^. Transaminase increases were infrequent, and most were low-grade and transient. Symptoms that may be suggestive of dorsal root ganglia toxicity were observed in four patients in total of 153 participants. Most serious adverse events were related to infection. In these two trials (NCT05386680 and NCT05089656), a total of 1.1E + 14 vector genomes were injected into the CSF of human patients weighing around 10-35 kg. The estimated CSF volume in humans and neonatal mice is 100-150 milliliters and 3-8 microliters, respectively. Therefore, the CSF vector concentration in our mouse experiments is 8.5 to 68-fold higher than in clinical conditions (0.6-5.0E + 13 vg/mL in mice vs 0.7-1.1E + 12 vg/mL in humans). Compared with the vector dose in our mouse studies, based on body weight (3.3E + 13 vg/kg in SMA mice and 1.0E + 14 vg/kg in WT mice), the SMA patients were treated at 9-32-fold lower doses (3.1E + 12-1.1E + 13 vg/kg in humans). The high vector dose in our mouse studies may overestimate the safety concerns associated with the benchmark vector, including HCC risk. The future of SMA gene therapy should focus on optimizing the AAV capsid and therapeutic cargo to restore SMN function to physiologic levels. More efficient gene therapies will enable lower doses and allow a broader range of patients to receive safer, more effective treatment.

## Methods

### Vector design, construction, and production

Design and construction of the benchmark and 2nd-generation vector used in this study were described previously^22^. The benchmark (BMK) vector contains the *CMV* enhancer/*CB* promoter, an SV40 antigen intron, and the original human *SMN1* (ori-h*SMN1*) coding sequence. The 2nd-generation vector (2nd-G) contains the endogenous human *SMN1* promoter and codon-optimized *SMN1* coding sequence (opt-h*SMN1*). Both constructs contain a bovine growth hormone polyadenylation (pA signal). mTR and wtTR represent the mutated and wild-type inverted terminal repeat, respectively. This benchmark construct is similar in design to the AVXS-101 vector used in pre-clinical studies. Both vector genomes were packaged into AAV9 for in vivo evaluation. The rAAVs were produced by transient HEK293 cell transfection and CsCl ultracentrifugation by the UMass Chan Medical School Viral Vector Core, as previously described^90^. Vector preparations were titered by ddPCR, and purity was assessed by 4-12% SDS-PAGE and Flamingo staining (Invitrogen).

### Animal procedures

The animal study design follows the ARRIVE guidelines^91^. Animal experiments (protocol #: PROTO202000038) were approved by the Institutional Animal Care and Use Committee (IACUC) of UMASS Chan Medical School. C57BL/6 mice were purchased from Charles River. SMA mice (FVB.SMNΔ7; SMN2; Smn-, IMSR_JAX:005025) were purchased as breeding pairs from Jackson Laboratory. SMA parental mice were mated in the UMass Chan Medical School Animal Facility. Mice were housed in standard cages in a temperature-controlled room (22–24 °C) with a 12 h dark-light cycle and fed with standard chow (LabDiet, #5P7622; 22.5% protein, 5.4% fat, 4% fiber, 50% polysaccharide). Tail samples were collected from pups upon delivery, and DNA was extracted using KAPA lysis buffer (KAPA Express Extract, Roche, 07961626001) from each sample. After genotyping, each litter was culled to three pups per cage. SMA or healthy control animals were injected with the indicated dose of AAV vector at a total volume of 4 µL (2 µL per side) via ICV injection. SMA mice were randomly divided into different groups, and age-matched healthy carriers were used as controls. Both male and female mice were used in the study. This study was not conducted in a double-blind manner. The behavior testing, including righting reflex, grid latency, and rotarod performance, was performed at the indicated time points as previously described^22^. The SMA mice will be euthanized if they cannot right themselves within 30 seconds. The C57BL/6 will be euthanized if the body weight loss is >20%. All studies were performed in compliance with relevant ethical regulations.

### Echocardiography

Echocardiography tests were performed at the UMass Chan Medical School Cardiovascular & Surgical Models Core, in accordance with IACUC protocol #20220006. Depilatory cream was applied to the abdomen and chest of each animal, then animals were induced with 2.0% isoflurane mixed with 0.5 L/min of 100% O_2_ and gently affixed to a heated physiologic platform of the Vevo 3100 imaging system (Visual Sonics, Toronto, ON, Canada). Electrode cream was applied to each limb. Body temperature was continually monitored and maintained at 37 °C with a rectal temperature probe. Isoflurane was administered by nose cone, then the concentration was reduced to 1.0% isoflurane mixed with 0.5 L/min of 100% O_2_. All animals were imaged at a heart rate of at least 450–500 beats per minute. 2-D and M-mode images were obtained in the parasternal long and short axes with a 50 MHz transducer (MX550S) as previously described^92,93^. Analysis was performed offline using Vevo LAB image analysis software.

### Hematoxylin and eosin (H&E) staining

Mice were perfused with pre-cooled Dulbecco’s PBS (DPBS) after anesthesia with isoflurane, then perfused with cold 4% paraformaldehyde (PFA) in DPBS. The tissues were isolated and stored in 4% PFA at 4 °C overnight. Tissues were embedded in paraffin and sectioned into 5 µm slices with a Thermo Shandon Finesse ME+ Microtome. The UMass Chan Medical School Morphology Core performed paraffin embedding and H&E staining. Images were captured using a Leica Thunder DMi8 microscope system.

### Western blot

Tissues were frozen with liquid nitrogen and ground into fine powders, then transferred to a 1.5 mL tube containing 250 µL protein lysis buffer: T-PER™ Reagent (Thermo Scientific, 78510) and RIPA (Thermo Fisher Scientific,89900) with protease Inhibitor Cocktail (Millipore Sigma, P8340) and incubated on ice for 30 minutes. DRG samples were homogenized in 2.0 mL screw cap tubes containing 100 µL protein lysis buffer using the OMNI Bead Ruptor Elite. Lysates were centrifuged at 12,000 rpm for 10 minutes, then the supernatant was transferred into a fresh 1.5 mL tube. Bicinchoninic acid (BCA) assay was used to measure the protein concentration. 4x Laemmli Sample Buffer (Bio-Rad, 1610747) was added to protein lysates, then samples were heated at 100 °C for 3 minutes and centrifuged at 8000 rpm. After that, samples were stored at −80 °C or immediately loaded into SDS-PAGE gels for Western blotting. Mouse serum was diluted 10-fold with DPBS, then added to an appropriate volume of 4× loading buffer and heated at 100 °C before loading into SDS-PAGE gels. Protein lysates were separated using an 8–16% Mini-protein TGX gel (Bio-Rad, Cat#: 4561106) and transferred onto a TransBlot Turbo Midi-size nitrocellulose Membrane (BioRad Trans-Blot Turbo RTA Transfer Kit. Cat#:1704271) using Trans-Blot Turbo. Membranes were blocked in Intercept (PBS) Blocking Buffer (Li-Cor, 927-70001). Membranes were incubated with a mouse anti-SMN antibody (Fisher Scientific, BDB610646) and rabbit anti-Gapdh antibody (Thermo Scientific, MA5-44674) at 4 °C for 8–10 hours. Membranes were then washed three times with 1× Tris Buffered Saline (TBS)-Tween (Boston BioProducts, IBB-181X). Then, IRDye 680-conjugated goat anti-mouse IgG polyclonal (LI-COR Biosciences, 926-68070) and IRDye 800CW-conjugated goat anti-rabbit IgG (LI-COR Biosciences, 926-32211) secondary antibodies (1:2,000) were diluted in the blocking buffer and incubated with the membranes for 1–2 hours. Membranes were then washed three times with TBST and imaged with an Odyssey CLX (Li-Cor). Quantification analysis was done using the ImageJ open-source software.

### Immunofluorescence staining on frozen samples

Animals were perfused with PBS to flush out the blood, followed by a 4% formaldehyde solution to fix the tissues. The DRG and spinal cord were isolated and fixed by immersion in a 4% PFA solution (DRG, 2–4 hours; spinal cord, 4–8 hours), then immersed in a 15% sucrose solution overnight at 4 °C, then transferred to 30% sucrose overnight to dehydrate the tissue. Spinal cord samples were embedded in diluted Optimal Cutting Temperature (OCT), which was diluted with 30% sucrose at a 1:2 ratio of OCT:30% sucrose. The lumbar DRGs were embedded with yellow Polyfreeze Tissue Freezing Medium (SHH0024-6X120ML, Sigma-Aldrich). Cryosections were cut at a thickness of 25 µm within the lumbar L1 and L5 segments and mounted onto gelatin-coated histological slides. Spinal cord sections were incubated with 0.2% Triton X-100 (diluted with PBS) for 15 minutes at room temperature and blocked with 10% donkey serum in TBS (LI-COR, 927-60001), DRG sections were blocked with a solution of 10% donkey serum, 10% goat serum, and 0.2% Triton X-100 in TBS for 1.5 hours, then incubated with anti-ChAT (1:200, Sigma-Aldrich, HPA048547), anti-SMN (1:200, Fisher Scientific, BDB610646), or anti-Parvalbumin (1:1000, ab11427, Abcam), anti-SmB (1:50, sc-130670 AF594, Santa Cruz Biotechnology) antibodies overnight at 4 °C. Slides were washed with PBS three times for 15 minutes each and then incubated with fluorescent-conjugated goat anti-mouse 488 (1:1000, A11029, Thermo Fisher Scientific), donkey anti-rabbit 647 (1:1000, A-31573, Thermo Fisher Scientific) antibodies, as well as Hoechst 33342 (1:2000, #62249, Thermo Scientific) for 2 hours at room temperature. After three washes with PBS, the slides were mounted with Fluoromount-G Mounting Medium (0100-01, Southern Biotech) for imaging with a Leica SP8 microscope.

### Motor neuron quantification

The spinal cord was immersed in 4% formaldehyde solution for 14-16 hours to fix, then transferred to 30% sucrose overnight. Specific L1-L5 lumbar segments were identified based on the ventral roots, and segmental identity was further confirmed by characteristic ventral horn morphology. Lumbar segments were embedded with OCT:30% sucrose (1:2) solution. The serial transverse sections were cut at 30 μm thickness with a cryostat, and sections were collected from the central portion of each segment, avoiding transitional boundaries between adjacent segments. Around 35 sections were obtained from each L1 and L5 segment. Motor neurons were quantified from L1 and L5 regions using systematic random sampling. To avoid double-counting of the same neurons across adjacent sections, every four-fifths section was selected for immunostaining and analysis, starting from a randomly chosen initial section. Seven evenly spaced sections per segment in each animal were selected to provide a representative sampling of motor neuron populations. All the chosen slides were stained with Hoechst 33342 and anti-ChAT (1:100, EMD Millipore AB144P). ChAT-positive neurons with a clearly identifiable nucleus located in the ventral horn were counted^94–96^.

### Immunofluorescence staining on paraffin-embedded samples

Lumbar segments were fixed in 4% PFA overnight, embedded with paraffin, and then sectioned at 5 μm thickness with 45 μm intervals. Slides were deparaffinized, rehydrated, and incubated with antigen retrieval buffer (Antigen Unmasking Solution, Citric acid-Based pH 6). This process was completed in a pressure cooker at 110 °C for 15 minutes. Slides were allowed to cool for 20 minutes until reaching room temperature by slowly adding water to the retrieval container. Once slides were cooled to room temperature, they were washed for 5 minutes each in TBS, TBS, then TBS+Tween20 (TBS). Sections were blocked with a 2.5% normal goat serum for VGluT1 or 3% BSA for ChAT staining, incubated at room temperature for 20 minutes, and then incubated with the primary anti-VGluT1 (1:200 diluted in 2.5% normal goat serum; Synaptic Systems, 135-318) or anti-ChAT (1:100 diluted in 3% BSA; Sigma-Aldrich, AB144P) at 4 °C for 10–12 hours. Donkey anti-goat IgG (H + L) Alexa Fluor Plus 555 (Cat # A32816; LOT: XB334919) and goat anti-rabbit IgG (H + L) Alexa Fluor Plus 647 (Cat # A32733; LOT: YG374179) secondary antibodies were then applied and allowed to incubate for 1 hour at room temperature, then slides were washed three times with TBS. All slides were stained with Sudan Black for 20 minutes to quench background and autofluorescence, washed under running water, and counterstained with 4’,6-diamidino-2-phenylindole (DAPI) for imaging with a Leica SP8 microscope.

### Immunohistochemistry (IHC) analysis

Paraffin cassettes were cut at 5 μm/section, deparaffinized and rehydrated, and then immersed in water. All slides were treated with antigen retrieval buffer (Vector Laboratories; H-3300-250 or Antigen Unmasking Solution). After antigen retrieval, slides were cooled to room temperature and were washed three times for five minutes each with TBS, TBS, and TBST sequentially. Slides were then blocked with 2.5% normal horse serum for 30 minutes and incubated with primary antibodies: anti-AFP (sab3500533, Sigma Aldrich), anti-EpCAM (42515, Cell Signaling Technology), and Ki-67(ab16667, Abcam) overnight at 4 °C. Slides were then washed three times with TBS, then incubated with the corresponding secondary antibody for 1 hour. Then, ImmPACT DAB EqV (Vector Labs; SK-4103-400) was used to visualize the signal.

### Immunofluorescence staining and quantification of NMJs

Animals at P11 were perfused with cold PBS, and the QL muscle was fixed with 4% PFA for 15 minutes at room temperature. Muscles were washed with PBS three times, then the QL muscle was immersed in a 30% sucrose solution overnight at 4 °C and embedded in OCT:30% sucrose solution for cryostat sectioning (30 μm). Slides were washed with BPS for 5 minutes after cryosection, rinsed in 2% Triton X-100 in PBS for 30 mins, then blocked with blocking buffer (0.3% BSA in 0.1% Triton X-100 in PBS) for 1.5 hours at 4 °C. Then incubate overnight at 4 °C with rabbit anti-Neurofilament (1:100, EMD Millipore, AB1987) and guinea pig anti-Synaptophysin (1:100, Synaptic Systems, 101 308), then incubate with secondary antibodies (1:1000) and alpha-Bungarotoxinin conjugates AF594 (1:200, BTX, Thermo Scientific, B13423). All slides were mounted with Fluoromount-G Mounting Medium for microscope imaging. The NMJ innervation analysis was done in a double-blind way using the same method described previously^21,47^.

### Electrophysiology

All animals were anesthetized with a combination of ketamine, at a dose of 37.5 mg/kg, and dexmedetomidine, at a dose of 0.5 mg/kg, administered intraperitoneally. Animals were kept on hand warmers throughout the procedure to maintain body temperature. A Natus® Nicolet EDX system was used with an AT2 + 6 amplifier and Natus® Viking Elite software. For electromyography a Natus® Ultra Disposable Subdermal Needle Electrode (cat.019-476600) was placed on the dorsum at the level of the first thoracic vertebra for grounding, and a Natus® TECA® Elite Disposable Concentric EMG Needle Electrode (cat. S53153) was used for a combination of reference and active/recording. For nerve conduction testing, three pairs of Natus® Ultra Disposable Monopolar Needle Electrodes (cat. 027721) were placed for stimulation along multiple points of the nerve; one set was at the level of the hip, one set was at the level of the stifle, and one set was at the level of the hock. Two subdermal needle electrodes were placed, the ground in the calcaneal tendon, and the reference was placed in the distal palmar aspect of the paw. A monopolar needle electrode was placed in the interdigital muscle of the paw. The nerve stimulation duration was set to 0.2 msec, and the intensity was supramaximal, defined as 50% above the stimulation needed to achieve a maximal compound muscle action potential (CMAP) amplitude. The distance between the stimulation sites was measured manually with a digital caliper. The waveform markers were placed manually as follows: 1 was placed at the point in which the waveform first deviated from baseline, 2 was placed at the peak of the first phase of the waveform, 4 was placed at the peak of the second phase of the waveform, and 5 was placed at the point of return to baseline. Waveform latency is the time from the stimulus to point 1 and was calculated automatically by the machine. The amplitude 2-4 of all waveforms was calculated by the machine in mA. The area under the curve of each waveform was calculated by the machine.

### PCR for AAV integration analysis

Total genomic DNA was isolated from liver or liver tumors with QIAamp DNA Mini Kit (51306, Qiagen), followed by quality and concentration assessment using NanoDrop One (Thermo Scientific). A total of 51 forward primers were designed against sequences in the mouse genomic *Rtl1*-*Rian* locus on chromosome 12, and three reverse primers were designed to target the AAV vector genome. DNA samples from liver tumors were used as templates for amplification using *OneTaq* 2X Master Mix (M0486L, New England Biolabs). Tumor-adjacent liver tissues from mice treated with the benchmark vector were used, and healthy liver tissues from WT or 2nd-generation vector-treated mice were used as comparisons. A total of 153 PCR reactions were conducted using DNA templates from tumor or non-tumor tissues. After the first round of PCR, the primer pairs that generated PCR products were used for a second round of PCR using genomic DNA from healthy liver or liver tumor samples. PCR reactions were denatured at 94 °C for 30 seconds, followed by 35 cycles of 94 °C for 20 seconds, 59 °C for 30 seconds, and 68 °C for 2 minutes, then a final extension step of 68 °C for 5 minutes. The resulting PCR products were TOPO cloned for Sanger sequencing. The primer sequences are shown in Supplementary Table 1.

### RNA analysis

The expression of microRNAs, lncRNAs, snoRNAs, and mRNAs was quantified by ddPCR. Firstly, total RNA was isolated using TRI Reagent (R2050-1-200, Zymo Research) from liver tissue or liver tumor and reverse transcribed by Taqman™ Advanced miRNA cDNA Synthesis Kit (Thermo Scientific, A28007). Homogeneous droplets were generated by the Automated Droplet Generator Instrument (Bio-Rad), and PCR products were analyzed in QX200 and QX600 Droplet Reader (Bio-Rad). Taqman assay-based primers with probes were purchased from Thermo Scientific: *Dlk1* (Mm00494477_m1, 4331182), *Rtl1* (Mm02392620_s1, 4331182), *Mirg* (Mm01335848_m1, 4351372), *Rian* (Mm01325842_g1, 4351372), miR-1188 (Mm04240859_s1, 4426961), miR-134 (Mm04238117_s1, 4426961), miR-543 (Mm04238293_s1, 4426961), miR-300 (Mm04238158_s1, 4426961), miR-487b (Mm04238299_s1, 4426961), miR-382 (Mm04238260_s1, 4426961), and *Tbp* (Mm01277042_m1, 4448484). The primers/probes for snoRNAs were synthesized by Integrated DNA Technologies (IDT). The primer/probe sequences are shown in Supplementary Table 2.

### RNA sequencing and bioinformatics

Strand-specific RNA-Seq libraries were prepared as previously described^97^. Paired-end RNA-Seq reads were first aligned to ribosomal RNA (BK000964.1) with Bowtie2^98^. Non-rRNA reads were subsequently used for gene quantification with Salmon v0.8.2^99^. Differential analysis was performed using DESeq2 v1.46^100^. DESeq2 was used to calculate *P* values by modeling read counts with a negative binomial distribution and applying Wald tests to assess differential gene expression between experimental conditions.

### Statistical analysis

Survival curve analyses were performed using the Log-rank (Mantel-Cox) test. The unpaired two-sided Student’s *t*-test was performed to compare two groups, and a one-way analysis of variance (ANOVA) test followed by the appropriate *post hoc* test was used to compare more than two groups. The percentage of frequency of peripheral tissue disease manifestations, cardiac thrombosis, liver tumor, and lung tumor was determined by a two-sided Fisher’s exact test. All data were analyzed by GraphPad Prism 10, and values are shown as mean ± standard error of the mean, unless otherwise specified. All data shown are biological replicates from distinct samples.

### Reporting summary

Further information on research design is available in the Nature Portfolio Reporting Summary linked to this article.

## Supplementary information

Supplementary Information

Reporting Summary

Transparent Peer Review file

## Source data

Source Data

## Data Availability

The gene expression data reported here have been deposited in the NCBI Sequence Read Archive (SRA) under the accession number: PRJNA1365939. Source data are provided with this paper.
