# Supplementary material for: Long-term comparative analysis of AAV9-mediated gene replacement therapies for spinal muscular atrophy in mice
Source: Nat Commun. 2026 May 23;17:6767. doi: 10.1038/s41467-026-73545-8 (PMC13385853; doi:10.1038/s41467-026-73545-8)
Supplement: Supplementary file 1 — Supplementary Information [file 41467_2026_73545_MOESM1_ESM.pdf]

Supplementary Table 1. PCR primers for integration analysis

Name	Orientation	Sequence (5′–3′)
R(a)	Reverse	CACTTGATGTACTGCCAAGTG
R(b)	Reverse	CCTCGAGTAGATGTACTGCC
R(c)	Reverse	GGCCATTTACCGTAAGTTATGTAAC
F(1)	Forward	CTAAGTCACCATGGAGAACCAG
F(2)	Forward	GAAGTGAAATCACAGCCTAGG
F(3)	Forward	ACTGGACACACTGTGCTTAAG
F(4)	Forward	CTCAAGCCTGTATTTCATCCC
F(5)	Forward	CTCTCTTCAGGGTCCTCTCT
F(6)	Forward	TCTGTGTATTGACTGCAGTCTAG
F(7)	Forward	CTGTTAAGCGTTTCTCTGGAC
F(8)	Forward	CTGCTCACTTTCTGACTGTATC
F(9)	Forward	TGTTACTCTGTCAGCCCATAC
F(10)	Forward	CATGTCAAGCTGTATATCTTCAGC
F(11)	Forward	GTGAATGGAGACTAGCCTGC
F(12)	Forward	CATGCAAACACAGTGCTGTG
F(13)	Forward	GGGTACCTCTAGTGTCAAGAGT
F(14)	Forward	CACATAGTGCAGATATTAGCTAGC
F(15)	Forward	CCTTGTCACTGATAACACTTAGAGG
F(16)	Forward	AAGACTGTCTGTGCTGCTAC
F(17)	Forward	CAATGATTATGATTAACCCTGTCGC
F(18)	Forward	CTCTTTGAATTTCTAAATCTTGCTAAGTG
F(19)	Forward	CTGTCTGAGTCAACACTTTTGG
F(20)	Forward	GACTCACAGACACACATAGACA
F(21)	Forward	CTCATCATATGGTAGTCCATTGTG
F(22)	Forward	CTAGTCAATTGGAAAAGGGATTCTT
F(23)	Forward	GAGAGAAGTAGATAGTTACAGTACCAAC
F(24)	Forward	GAGAAATGACAGAGAGTCACAGG
F(25)	Forward	GTTGTGGTTATATTGTTCAGTTAGTATGT
F(26)	Forward	TCTCAAGCTGTCTTCCCTTTC
F(27)	Forward	GTCCATATTCTGCTGTCGCTTC
F(28)	Forward	TGTGTGAGTATCATCAGCGTG
F(29)	Forward	ACATTCAGGTCTAATATATACTGAAGAATC
F(30)	Forward	GTTTCTTCCAGTCAAGACATCC
F(31)	Forward	GGGTCAGAGCCATGAAATAGTATAG
F(32)	Forward	CCTTTCCGATTGTGAAAGTGTG
F(33)	Forward	CTTCGCTTCTATTGTTGCAATTTC
F(34)	Forward	GGGAAACTGTAACACAAATACATTGTA

Name	Orientation	Sequence (5′–3′)
F(-1)	Forward	CCACTTTACCCATTGCAAACAG
F(-2)	Forward	GTATGGATGGACATGGAGCA
F(-3)	Forward	CATTCATTCTACAAGCTTATCTCCCAC
F(-4)	Forward	GATCATGTAAGGGATTAGATATTTCTGC
F(-5)	Forward	GTTCCCTTCCCATTTGTAGTCA
F(-6)	Forward	GATGAGTTGATTGTTTCATCCATGC
F(-7)	Forward	GTCAGTCAGCATGTCATACAGTA
F(-8)	Forward	CTCTCAACATCACCAGGACAG
F(-9)	Forward	CATGCTCCATGTTCTCCAGT
F(-10)	Forward	GTCACCAGCTCTCTTCACAG
F(-11)	Forward	GAGTCTGTCCTACCTGTTGTC
F(-12)	Forward	TGACTCTGAGCTCCTCCATC
F(-13)	Forward	TCATGCAGCTGTCCCTTATC
F(-14)	Forward	CATCGTGTAGATGGACGTTGC
F(-15)	Forward	TGGTTTGTGTAAGTATGAGTCCTG
F(-16)	Forward	CTAGTATTGTGAGGCTCTGGG
F(-17)	Forward	CTAGTCTCCAGTGATGATCAAAGG

Supplementary Table 2. Primer/probe for droplet digital PCR

Name	Orientation	Sequence (5′–3′)
MBII-19 (AF357341)	Forward	GGACCTATGATGAGATCTGGT
	Reverse	CCTGACCTCAGACATCTGTT
	Probe	CATGGCTGTCATCGAGATACAGTCAGATGC
MBII-49 (AF357426)	Forward	GGACCAATGATGACCTTGT
	Reverse	ACCTCAGAATCCAGTATGTTG
	Probe	TCTATATTATAAGTCATAGACGATGAC
MBII-343 (Gm26922)	Forward	TGGATCTGTGATGACAAAGG
	Reverse	GAATCTCAGACTTCCAGACAT
	Probe	TACTCATGATCCACAACTCATACGCCA
MBII-78 (AF357428)	Forward	AGGACCGATGATGAGATC
	Reverse	CCTGACCTCAGATATCTGTT
	Probe	ATGTCATCGAGAAACACTCAGACACCA
MBII-48 (AF357425)	Forward	TAGGCCAATGATGAGGAG
	Reverse	TCCTCAGATTTCATAAGGGTTTAA
	Probe	CACTGTCCTCGGTCAGAAACCCCA
miR-16	Forward	GTCAGCGGTGCCTTAGCA
	Reverse	CCTTACTTC AGCAGCACAGTC
	Probe	GGAGGTAATTTCAGAATCTTAACGCCAATA
U6	Forward	CGCTTCGGCAGCACATAT
	Reverse	AACGCT TCACGAATTTGCGT
	Probe	CGCAGGGGCCATGCTAATCTTCTCTGT
SMN	Forward	CACCCGCGGGTTTGCTATG
	Reverse	TCATCGCTCTGGCCTGT
	Probe	CCACTGCCGCCGCTGCTCAT

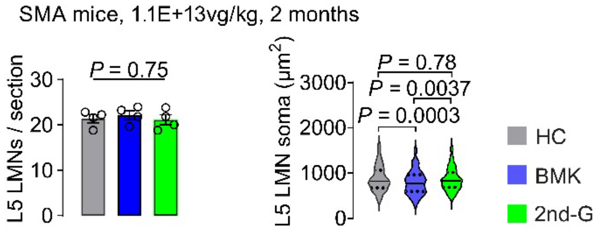

Supplementary Figure 1. Neuron number and soma size of L5 LMNs in the SMA mice treated with AAV at 1.1E+13 vg/kg for two months. Healthy carriers are used for comparison. To calculate soma size, ~70 motor neurons were sampled from each mouse. N = 4 mice in each group. One-way ANOVA followed by Tukey's multiple comparisons was used for statistical analysis; error bars are presented as mean ± SEM.

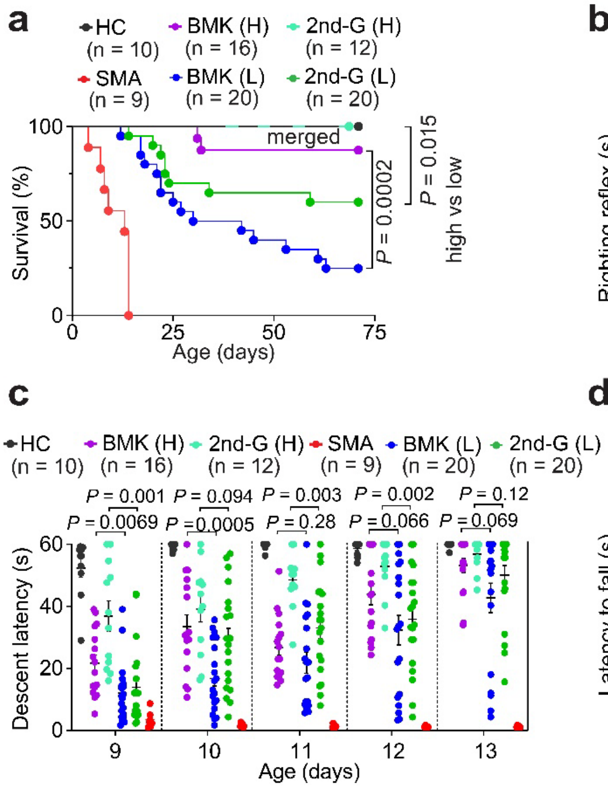

Supplementary Figure 2. Dose responses of the 2nd-generation and benchmark vectors on the SMA mice. SMA mice were treated by ICV injection at P0 with two different doses: 1.1E+13 vg/kg (L) and 3.3E+13 vg/kg (H). Survival (**a**), righting reflex (**b**), grid test (**c**), and rotarod test (**d**) were measured at the indicated study period or days. Log-rank (Mantel-Cox) test in (**a**) and unpaired Student's *t* tests were applied in (**b**, **c**, **d**). Error bars presented as mean ± SEM, each dot point represents an individual mouse in (**c**, **d**).

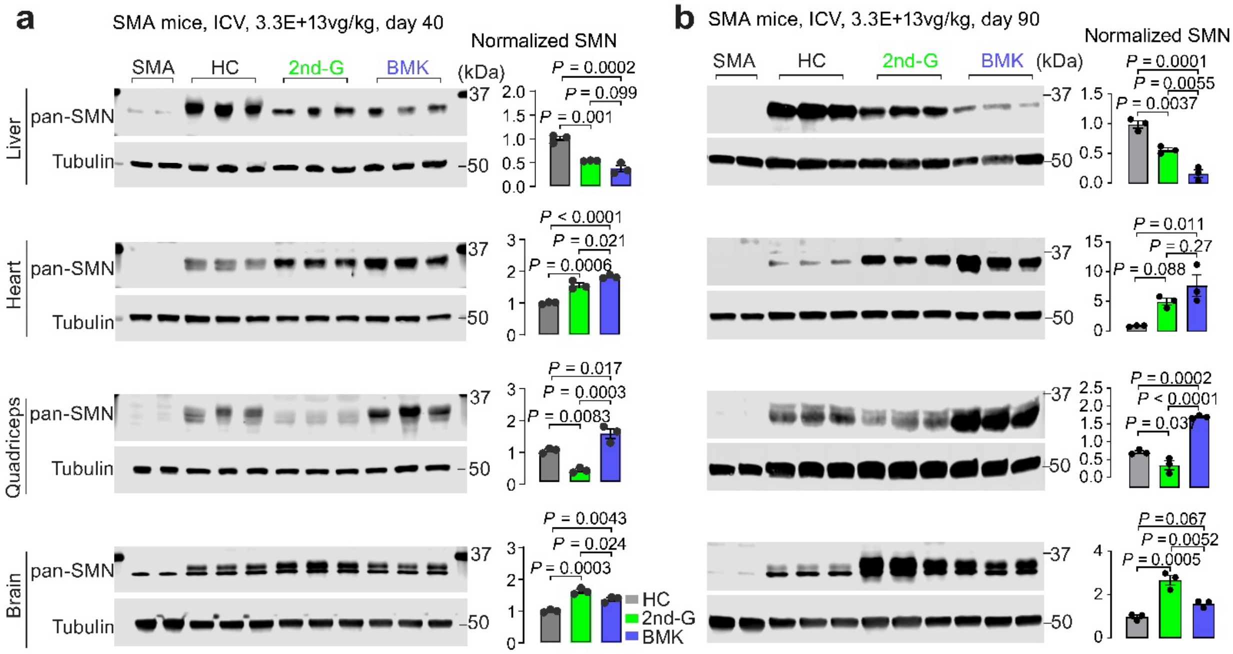

Supplementary Figure 3. SMN expression profile in the SMA mice after AAV treatment. SMA mice were treated with AAV9-SMN vectors at a dose of 3.3E+13 vg/kg via ICV injection at P0. Tissues were harvested at days 40 (**a**) or 90 (**b**) for Western blot analysis. One-way ANOVA followed by Tukey's multiple comparisons test were applied, error bars presented as mean ± SEM, n = 3 mice in each group.

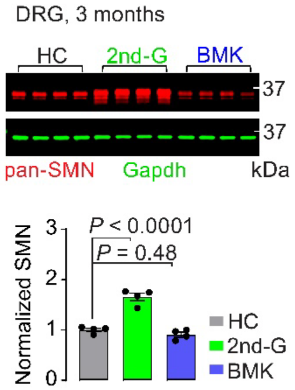

Supplementary Figure 4. Western blot analysis of SMN expression in the dorsal root ganglion (DRG) of treated SMA mice for three months. 46-48 DRGs/mouse were used for protein extraction for analysis. N = 4 mice in each group. One-way ANOVA followed by Tukey's multiple comparisons test. Error bars presented as mean ± SEM.

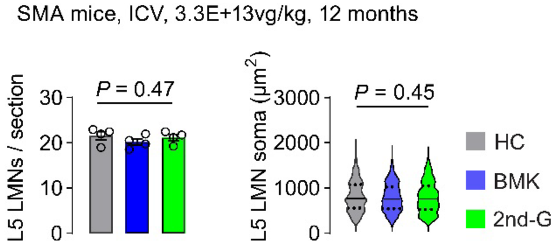

Supplementary Figure 5. Neuron number and soma size of L5 LMNs in the SMA mice treated with AAV at 3.3E+13 vg/kg for 12 months. Healthy carriers are used for comparison. To calculate soma size, ~120 motor neurons were sampled from each mouse. N = 4 mice in each group. One-way ANOVA followed by Tukey's multiple comparisons was used for statistical analysis; error bars are presented as mean ± SEM.

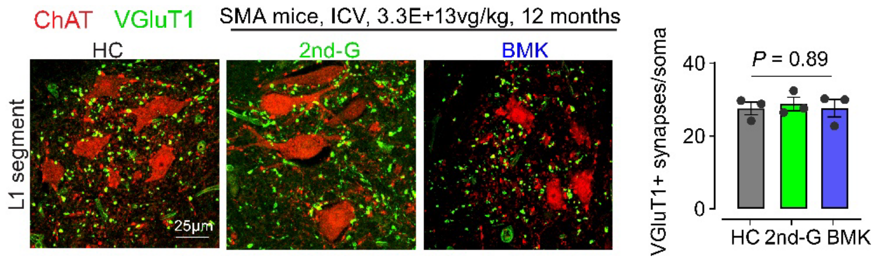

Supplementary Figure 6. Immunostaining of vesicular glutamate transporter 1 (VGluT1) and ChAT in the L1 lumbar segment of the spinal cord of SMA mice 12 months post-injection. One-way ANOVA followed by Tukey's multiple comparisons test. Error bars presented as mean ± SEM. N = 3 mice in each group.

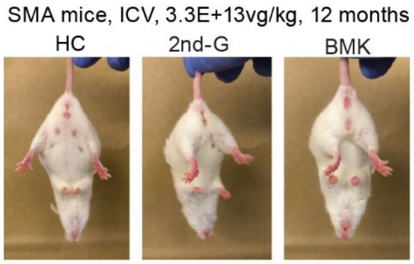

Supplementary Figure 7. No hind-limb clasping was observed in the SMA mice that received AAV for 12 months at 3.3E+13 vg/kg.

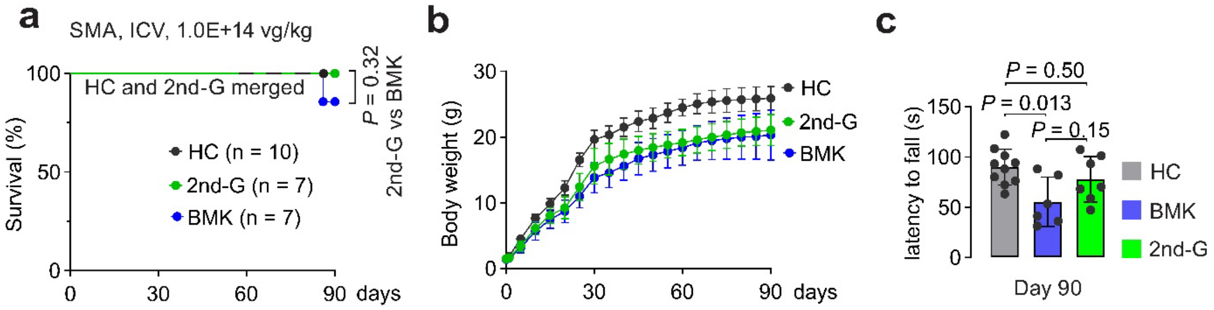

Supplementary Figure 8. Comparison of the therapeutic outcomes of the 2nd-generation and benchmark vectors on the SMA mice. SMA mice were treated by ICV injection at P0 with 1.0E+14 vg/kg. Survival curve (**a**), Bodyweight gain (**b**), and rotarod test (**c**) were measured at the indicated days in a 90-day study period. Animal numbers in (**a** and **b**): HC (n = 10), 2nd-G (n = 7), BMK (n = 7) in each group, and each dot point represents an individual mouse in (**c**). Statistical test used was Log-rank (Mantel-Cox) test in (**a**), one-way ANOVA followed by Tukey's multiple comparisons was used for statistical analysis in (**c**), error bars presented as mean ± SD.

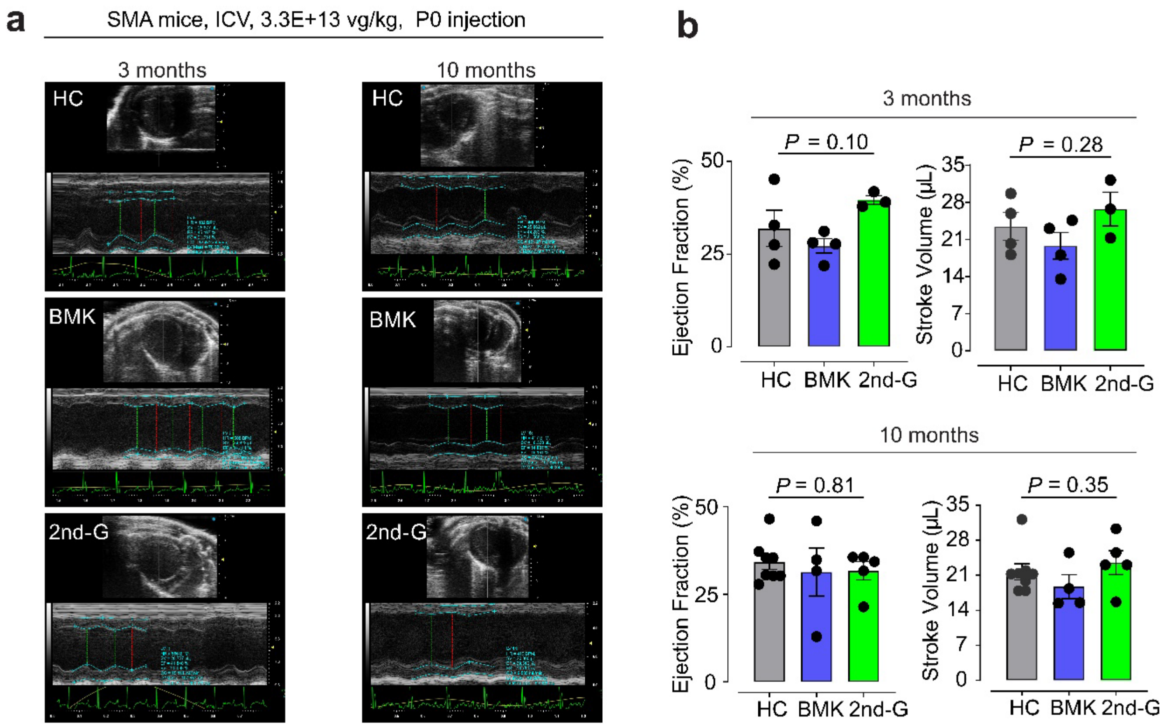

Supplementary Figure 9. 2D echocardiography (echo) assessment of heart function in the P0-treated SMA mice. a M-mode tracing of representative transthoracic echocardiograms of the left ventricle at 3- and 10-months post-treatment. **b** Ejection fraction (%) and stroke volume (μL) of the treated SMA mice at months 3 and 10. Healthy littermates are used for comparison. Each dot represents an individual animal. Data are present as mean ± SEM in (**b**). One-way ANOVA followed by Tukey's multiple comparisons test was performed.

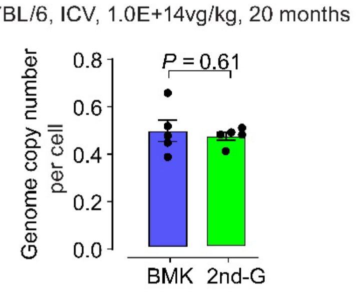

Supplementary Figure 10. AAV genome copy in the lumbar spinal cord segment. C57BL/6 wild-type mice were injected with 1.0E+14 vg/kg at P0 through ICV. After 20 months, genomic DNA was extracted from the lumbar spinal cord segment for ddPCR analysis. Unpaired Student's *t* test was used. Error bars presented as mean ± SEM. N = 5 mice in each group

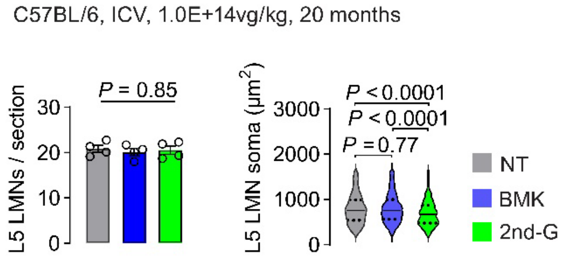

Supplementary Figure 11. Neuron number and soma size of L5 LMNs in the C57BL/6 mice treated with AAV at 1.0E+14 vg/kg for 20 months. Healthy carriers are used for comparison. To calculate soma size, ~170 motor neurons were collected from each mouse (n = 4 mice per group). One-way ANOVA followed by Tukey's multiple comparisons was used for statistical analysis, and error bars are presented as mean ± SEM.

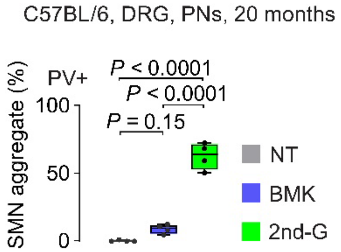

Supplementary Figure 12. SMN aggregates in DRGs of C57BL/6 mice treated with AAV at 1.0E+14 vg/kg for 20 months. To calculate the SMN aggregates, ~60 PV neurons were used per mouse (n = 4 mice per group). One-way ANOVA followed by Tukey's multiple comparisons was used for statistical analysis; error bars are presented as mean ± SEM.

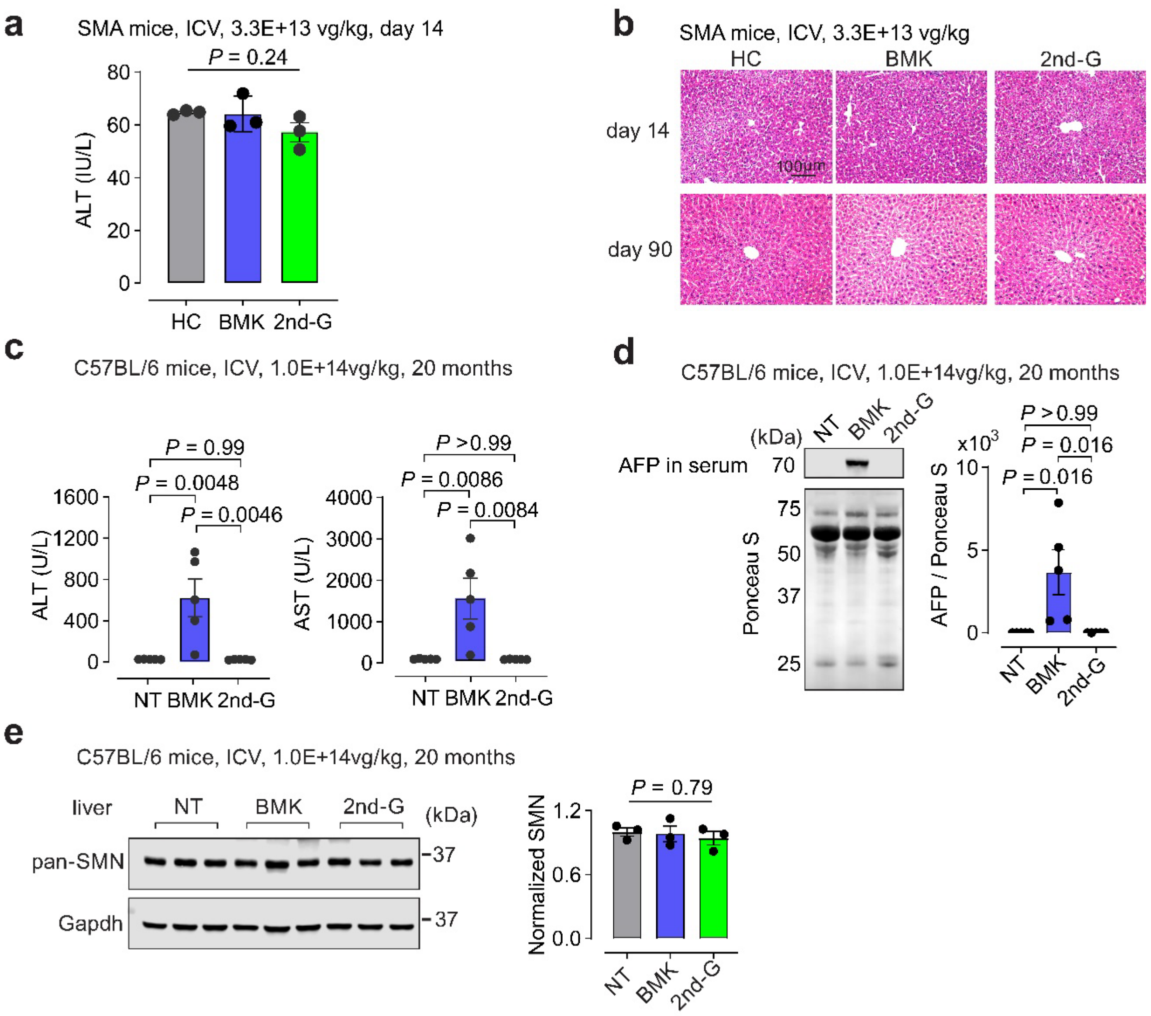

Supplementary Figure 13. Liver pathology after AAV-SMN gene therapy. (**a, b**) ALT level in serum and H&E staining in the liver. SMA neonates were injected with a dose of 3.3E+13 vg/kg at P0 through ICV. The ALT was measured on day 14. H&E staining was performed on days 14 and 90, n = 3 in each group. Scale bar = 100 μm. (**c)** Serum ALT and AST levels. C57BL/6 wild-type mice were injected with 1.0E+14 vg/kg at P0 via ICV. The serum was collected after 20 months post-injection for measurement. Each dot point represents an individual mouse. **d** Western blot analysis on Alpha-Fetoprotein (AFP) in serum from the C57BL/6 mice after 20-month treatment. Ponceau S solution staining was used to illustrate total protein; n = 5 mice per group. **e** SMN protein level in the liver of C57BL/6 wild-type mice after 20-month treatment. One-way ANOVA followed by Tukey's multiple comparisons test in all bar plots; error bars presented as mean ± SEM; n = 3 mice per group.

a

integration sites summary:

Chromosome 12, GRCm39	
tumor #	insertion site
HCC-1	109,581,648
HCC-2	109,577,985
HCC-3	109,577,964
HCC-4	109,583,710
HCC-5	109,577,973
HCC-6	109,566,767
HCC-7	109,566,767
HCC-8	109,583,710
HCC-9	109,583,710
HCC-10	109,577,987

b

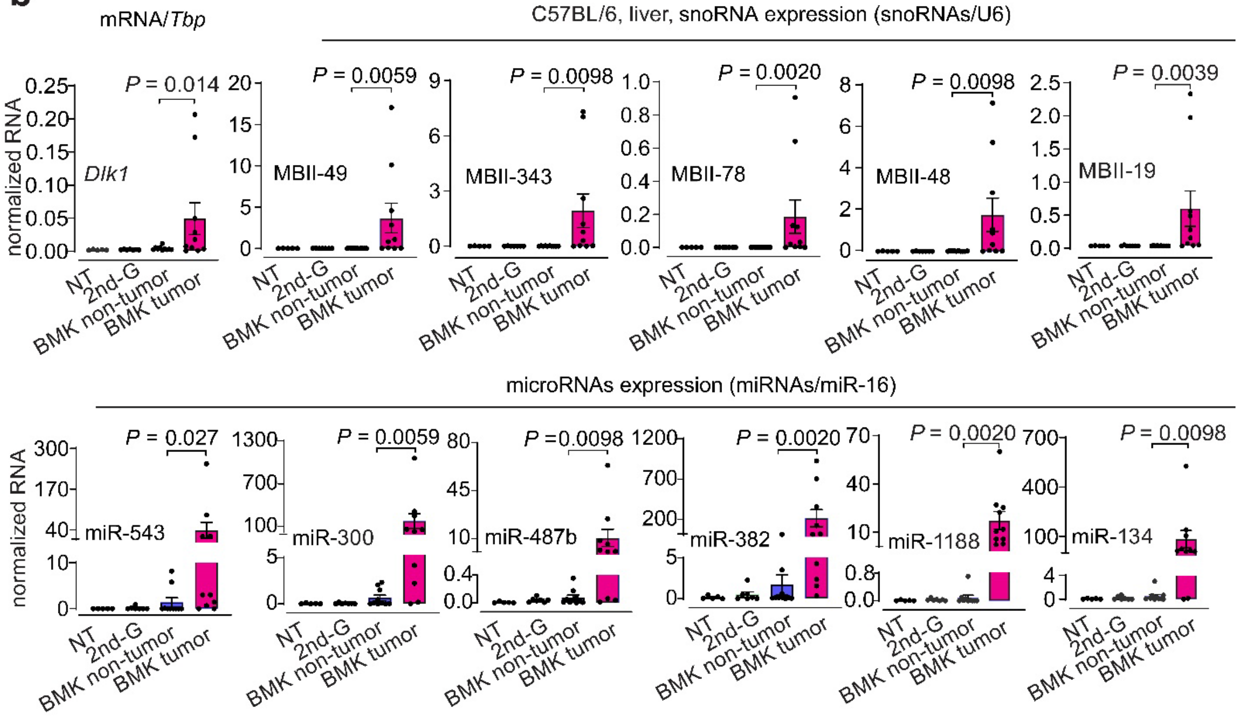

Supplementary Figure 14. BMK vector integrates into the mouse *Rtl1-Rian* locus and results in proximal non-coding RNA overexpression in HCC. a AAV genome integration sites on Chromosome 12 from BMK-induced HCC. **b** ddPCR analysis of mRNA and non-coding RNAs related to AAV vectors integration (n = 5 in NT, n = 7 in 2nd-G, n = 10 in BMK non-tumor, and n = 10 in BMK tumor). Wilcoxon matched pairs with the signed rank test was applied for data analysis; error bars presented as mean ± SEM.
