# Supplementary material for: Long-term comparative analysis of AAV9-mediated gene replacement therapies for spinal muscular atrophy in mice
Source: Nat Commun. 2026 May 23;17:6767. doi: 10.1038/s41467-026-73545-8 (PMC13385853; doi:10.1038/s41467-026-73545-8)
Supplement: Supplementary file 2 — Reporting Summary [file 41467_2026_73545_MOESM2_ESM.pdf]

nature portfolio

Corresponding author(s): Jun Xie

Last updated by author(s): Dec 8, 2025

Reporting Summary

Nature Portfolio wishes to improve the reproducibility of the work that we publish. This form provides structure for consistency and transparency in reporting. For further information on Nature Portfolio policies, see our Editorial Policies and the Editorial Policy Checklist.

Statistics

For all statistical analyses, confirm that the following items are present in the figure legend, table legend, main text, or Methods section.

n/a	Confirmed	
☐	☒	The exact sample size (*n*) for each experimental group/condition, given as a discrete number and unit of measurement
☐	☒	A statement on whether measurements were taken from distinct samples or whether the same sample was measured repeatedly
☐	☒	The statistical test(s) used AND whether they are one- or two-sided<br>*Only common tests should be described solely by name; describe more complex techniques in the Methods section.*
☐	☒	A description of all covariates tested
☐	☒	A description of any assumptions or corrections, such as tests of normality and adjustment for multiple comparisons
☐	☒	A full description of the statistical parameters including central tendency (e.g. means) or other basic estimates (e.g. regression coefficient) AND variation (e.g. standard deviation) or associated estimates of uncertainty (e.g. confidence intervals)
☐	☒	For null hypothesis testing, the test statistic (e.g. *F*, *t*, *r*) with confidence intervals, effect sizes, degrees of freedom and *P* value noted<br>*Give P values as exact values whenever suitable.*
☒	☐	For Bayesian analysis, information on the choice of priors and Markov chain Monte Carlo settings
☒	☐	For hierarchical and complex designs, identification of the appropriate level for tests and full reporting of outcomes
☒	☐	Estimates of effect sizes (e.g. Cohen's *d*, Pearson's *r*), indicating how they were calculated

Our web collection on statistics for biologists contains articles on many of the points above.

Software and code

Policy information about availability of computer code

Data collection	Odyssey M (Li-Cor) (for western blot image acquisition), LEICA SP8 software (v2.5.2.6939) (for confocal image acquisition), Vevo 3100 imaging system (Visual Sonics, Toronto, ON, Canada) for echocardiography recording.
Data analysis	LI-COR Acquisition (Ver 1.2) was used to analyze Western blot. Image J 1.52s software was used to quantify SMN and Gapdh intensity. GraphPad Prism 10 was used to perform statistical analysis. Paired-end RNA-Seq reads were first aligned to ribosomal RNA (BK000964.1) with Bowtie2. Non-rRNA reads were subsequently used for gene quantification with Salmon v0.8.2. Differential analysis was performed using DESeq v3.5. Echocardiography test, 2-D and M-mode images were obtained in the parasternal long and short axes with a 50 MHz transducer (MX550S).

For manuscripts utilizing custom algorithms or software that are central to the research but not yet described in published literature, software must be made available to editors and reviewers. We strongly encourage code deposition in a community repository (e.g. GitHub). See the Nature Portfolio guidelines for submitting code & software for further information.

Data

Policy information about availability of data

All manuscripts must include a data availability statement. This statement should provide the following information, where applicable:

- Accession codes, unique identifiers, or web links for publicly available datasets
- A description of any restrictions on data availability
- For clinical datasets or third party data, please ensure that the statement adheres to our policy

NGS data of HCC was uploaded the NCBI's Sequence Read Archive (SRA), using Series accession number PRJNA1365939. Full uncropped western blots are available as Source data.

Research involving human participants, their data, or biological material

Policy information about studies with human participants or human data. See also policy information about sex, gender (identity/presentation), and sexual orientation and race, ethnicity and racism.

Reporting on sex and gender	No human participants in this study.
Reporting on race, ethnicity, or other socially relevant groupings	Not applicable.
Population characteristics	Not applicable.
Recruitment	Not applicable.
Ethics oversight	Not applicable.

Note that full information on the approval of the study protocol must also be provided in the manuscript.

Field-specific reporting

Please select the one below that is the best fit for your research. If you are not sure, read the appropriate sections before making your selection.

☒ Life sciences ☐ Behavioural & social sciences ☐ Ecological, evolutionary & environmental sciences

For a reference copy of the document with all sections, see nature.com/documents/nr-reporting-summary-flat.pdf

Life sciences study design

All studies must disclose on these points even when the disclosure is negative.

Sample size	Sample sizes were based on similar work in the field in order to support meaningful conclusions (PMID: 33795885, PMID: 28504671, PMID: 34825141, PMID: 21315257, PMID: 21968514). The sample size for each experiment is detailed in the figure legends
Data exclusions	No data was excluded from the analysis.
Replication	All the data in the manuscript were generated using at least three independent biological replicates.
Randomization	Experimental mice were randomly assigned to treatment groups and untreated controls, all mice with same age.
Blinding	Blinding to treatment conditions was performed when running behavioral assays, echocardiography tests, motor neuron quantifications and electrophysiology test.

Reporting for specific materials, systems and methods

We require information from authors about some types of materials, experimental systems and methods used in many studies. Here, indicate whether each material, system or method listed is relevant to your study. If you are not sure if a list item applies to your research, read the appropriate section before selecting a response.

Materials & experimental systems

n/a	Involved in the study	
☐	☒	Antibodies
☒	☐	Eukaryotic cell lines
☒	☐	Palaeontology and archaeology
☐	☒	Animals and other organisms
☒	☐	Clinical data
☒	☐	Dual use research of concern
☒	☐	Plants

Methods

n/a	Involved in the study	
☒	☐	ChIP-seq
☒	☐	Flow cytometry
☒	☐	MRI-based neuroimaging

Antibodies

Antibodies used

Primary Antibodies:
Mouse anti-SMN (IF, 1:200, WB 1:2000, Fisher Scientific, BDB610646); Goat anti-ChAT (IF, 1:100, Millipore AB144P); Rabbit anti-ChAT (IF, 1:200, Sigma-Aldrich, HPA048547); Rabbit anti-Parvalbumin (IF, 1:1000, ab11427, Abcam); Mouse anti-SmB (IF, 1:50, sc-130670 AF594 , Santa Cruz Biotechnology); Guinea pig anti-VGluT1 (IF, 1:200 diluted in 2.5% normal goat serum; Synaptic Systems, 135-318); Rabbit anti-AFP (IHC,1:200, sab3500533, Sigma Aldrich); Rabbit anti-EpCAM (IHC,1:200, #42515, Cell Signaling Technology); Rabbit Ki-67 (IHC,1:200, ab16667, Abcam); Rabbit anti-Gapdh (WB, 1:1000, Thermo Scientific, MA5-44674); Mouse Beta Tubulin (WB, 1:1000, Thermo Scientific, MA5-16308); Rabbit anti-Neurofilament (IF, 1:100, EMD Millipore, AB1987); Guinea pig anti-Synaptophysin (IF, 1:100, Synaptic Systems, 101 308); alpha-Bungarotoxinin conjugates AF594 (IF, 1:200, Thermo Scientific, B13423). Pierce™ Hoechst 33342 Solution (IF, 1:4000,Thermo Scientific, 62249).

Secondary Antibodies:
Goat anti-mouse 488 (IF, 1:1000, Thermo Fisher Scientific, A11029); Donkey anti-rabbit 647 (IF, 1:1000, Thermo Fisher Scientific, A-31573); Donkey anti-goat IgG (H+L) Alexa Fluor Plus 555 (IF, 1:1000, Cat # A32816; LOT: XB334919); Goat anti-rabbit IgG (H+L) Alexa Fluor Plus 647 (IF, 1:1000, Cat # A32733; LOT: YG374179); Goat anti-rabbit Alexa 488 (IF, 1:500,Thermo Scientific, A32731); Donkey anti goat IgG (H+L) Alexa Fluo 594 (IF, 1:500,Thermo Scientific, A11058); IRDye 680-conjugated goat anti-mouse IgG polyclonal (WB, 1:2000, LI-COR Biosciences, 926-68070); IRDye 800CW-conjugated anti-rabbit IgG goat (WB, 1:2000, LI-COR Biosciences, 926-32211);

Validation

The antibodies used in this study have been widely published and experimentally validated as described below.

The mouse anti-SMN (WB 1:2000, Fisher Scientific, BDB610646) has been previously validated in WT and SMN-deficient cells and tissue (PMID: 34825141; PMID: 33795885; PMID: 17895963; PMID: 23063131; PMID: 23967270; PMID: 24332368).

The mouse anti-SmB (sc-130670 AF594 , Santa Cruz Biotechnology); has been previously validated in WT and SmB-deficient cells and tissue (PMID: 34825141; PMID: 33795885; PMID: 17023415; PMID: 17895963; PMID: 22037760; PMID: 26063904; PMID: 30012555; PMID: 31570875).

The rabbit anti-Gapdh (Thermo Scientific, MA5-44674) was validated and confirmed in this study and manufacturer (https://www.thermofisher.com/antibody/product/GAPDH-Antibody-clone-PD00-07-Recombinant-Monoclonal/MA5-44674).

The anti-Choline Acetyltransferase (ChAT, Sigma-Aldrich, AB144P) has been published and validated (PMID: 34825141; PMID: 33795885; PMID:31851921, PMID:28504671, PMID:30012555, PMID:29281826).

The rabbit anti-Neurofilament (EMD Millipore, AB1987) was validated in this study and other publications (PMID: 34825141; PMID: 33795885; PMID:31851921, PMID:28504671, PMID:30012555, PMID:29281826).

The guinea pig anti-Synaptophysin (Synaptic Systems, 101 308) was validated in this study and other publications (PMID: 34825141; PMID: 33795885; PMID:31851921, PMID:28504671, PMID:30012555).

The α-Bungarotoxin (Thermo Scientific, B13423) was wildly used in NMJ staining, and was confirmed by publications (PMID: 24574468; PMID: 21778516; PMID: 24947322; PMID: 27464347).

The guinea pig anti-VGluT1 (Synaptic Systems, 135-318) has been confirmed by many studies (PMID: 34825141; PMID: 33795885;PMID: 21315257).

The mouse Beta Tubulin (Thermo Scientific, MA5-16308) was confirmed by many studies (PMID: 38224318; PMID: 38367663; PMID: 38724625).

The rabbit anti-AFP (sab3500533, Sigma Aldrich) was confirmed by many publications (PMID: 33657866; PMID: 37578396; PMID: 26809505).

The rabbit anti-EpCAM (#42515, Cell Signaling Technology) has been confirmed by (PMID: 37030630).

The rabbit Ki-67 (ab16667, Abcam) has been validated by many studies (PMID: 41053731; PMID: 41051621; PMID: 41048975).

Animals and other research organisms

Policy information about studies involving animals; ARRIVE guidelines recommended for reporting animal research, and Sex and Gender in Research

Laboratory animals	The SMNΔ7 mouse line (Smn+/–; SMN2+/+; SMNΔ7+/+) was purchased from the Jackson Laboratory (Jax stock: #005025), and as breeders to generate SMA mice (Smn–/–; SMN2+/+; SMNΔ7+/+) under FVB genetic background. The C57BL/6 were ordered from Charles River.
Wild animals	his study did not involve wild animals.
Reporting on sex	Mice of both males and females were used and assigned randomly in this study.
Field-collected samples	This study did not involve samples collected from the field.
Ethics oversight	All animal procedures was approved by the IACUC committee of Massachusetts Chan Medical School, and mouse work was performed in accordance with all relevant ethical regulations.

Note that full information on the approval of the study protocol must also be provided in the manuscript.

Plants

Seed stocks	No plants were used in this study.
Novel plant genotypes	*Describe the methods by which all novel plant genotypes were produced. This includes those generated by transgenic approaches, gene editing, chemical/radiation-based mutagenesis and hybridization. For transgenic lines, describe the transformation method, the number of independent lines analyzed and the generation upon which experiments were performed. For gene-edited lines, describe the editor used, the endogenous sequence targeted for editing, the targeting guide RNA sequence (if applicable) and how the editor was applied.*
Authentication	*Describe any authentication procedures for each seed stock used or novel genotype generated. Describe any experiments used to assess the effect of a mutation and, where applicable, how potential secondary effects (e.g. second site T-DNA insertions, mosiacism, off-target gene editing) were examined.*
