# Supplementary material for: Long-term comparative analysis of AAV9-mediated gene replacement therapies for spinal muscular atrophy in mice
Source: Nat Commun. 2026 May 23;17:6767. doi: 10.1038/s41467-026-73545-8 (PMC13385853; doi:10.1038/s41467-026-73545-8)
Supplement: Supplementary file 3 — Transparent Peer Review file [file 41467_2026_73545_MOESM3_ESM.pdf]

nature portfolio

Peer Review File

Long-term comparative analysis of AAV9-mediated gene replacement therapies for spinal muscular atrophy in mice

Corresponding Author: Professor Jun Xie

This file contains all reviewer reports in order by version, followed by all author rebuttals in order by version.

Version 0:

Reviewer comments:

Reviewer #1

(Remarks to the Author)
The manuscript entitled "Long-Term Comparative Analysis of AAV9-Mediated Gene-Replacement Therapies for Spinal Muscular Atrophy in Mice" by Chen and colleagues report on long-term severe side effects in pre-clinical models following a genetic delivery (Zolgensma-LIKE AAV) of the SMN gene that has been FDA approved in severe SMA patients. In addition, they use SMA mice to compare the effects of a second generation vector which appears to provide no side effects and better outcome in mutant mice. The severe side effects of the Zolgensma vector (assigned as "benchmark" vector in the study) include cardiac defects (thrombosis) but more alarmingly, tumors in liver and lungs in SMA mice after several months post-administration. This study is important and timely due to the ongoing therapy of SMA patients, particularly the severe form of Type 1 SMA patients. Intriguingly, the authors report on the benefits in SMA mice following the administration of a novel vector expressing a codon-optimized hSMN1 transgene driven by the endogenous hSMN1 promoter. They report no adverse effects of the tumorigenesis observed under the Zolgensma-like vector, which also shows a more robust benefit in motor behavior which was dose-dependent. However, administration of this vector in healthy (or wild type control) mice resulted in sensory toxicity after twenty months from its administration. The study provides important insights and sheds light into the long term potential toxicity of Zolgensma in SMA patients. The second-generation AAV is not novel since the same authors reported its benefits in 2024 (EMBO Mol Med). The novelty of the current study regarding this AAV is the route of administration; being intracerebroventricular (or ICV) compared to intravenous (or IV) reported previously in 2024. The study is well designed and for most of the experiments well executed with reasonable interpretations. However, several aspects of the study require additional attention to detail, further experimentation and new analysis since there are some serious concerns regarding the validity of their conclusions.
The major concerns are explained below:
1. There is a general problem with reporting statistical results. I urge the authors to pay particular attention to detail for all results. I amplify my concerns by raising the following issues:
i) in Fig.1d, the authors report the p values, but they don't mention which groups they compare; is it between the "benchmark" and 2nd generation AAV or is between 2nd generation and SMA mice?
ii) The comparison in Fig.1e is not valid because the authors compare the total number of observations, rather than the average number of observations per mouse. By doing so, the authors compare a high number of data points, but at the same time this is considered to be biased since due to pseudoreplication. The authors should compare the average value per each animal across the different groups. In addition, the authors state in the figure legend that this comparison was performed using one way ANOVA with Dunnetts' post hoc testing; however, they only report p value between "benchmark" and 2nd generation AAV. The authors are encouraged to report p values between controls (black data points) and the two AAV approaches. In this manner, the reader will be able to get a better understanding of how strong the latency of the two experimental groups were to the values from the control mice.
iii) In Fig.2, the authors compare the results between "benchmark" and the higher dose (3.3E+ 13vg/kg) of the 2nd generation AAV. However, this comparison is not informative nor valid. The authors should compare the results between the doses (the low one, ie 1.1E+13vg/kg and the higher one, ie 3.3E+ 13vg/kg). Without such statistical comparison the authors cannot conclude that the higher dose is better than the lower dose. This comparison should include % survival, righting reflex, latency to fall, descent latency (inverted grid) for at least one (but preferably two) time point(s).
iv) In fig.2d, the p value presented in the graph is confusing. It shows that comparison between HC and 2nd generation is p=0.5 (ie not significant) where comparison between HC and "benchmark" the p value is <0.001, which judging from the graph, this is simply not the case. The authors should recheck the analysis.
v) The authors need to report the number of mice per experimental group used, rather than the number of data points; for

instance, the motor neuron counts presented in three different instances, is unclear how many mice have been used (in figs 1h, 2f and 4d).

2. The method for assessing motor neuron survival is flawed. First, in Figs 1h, 2f and 4d, it is unclear whether the authors have included both LMC and MMC motor neurons. Irrespective, LMC motor neurons are considered resistant motor neurons (i.e. do not degenerate) under SMN deficiency conditions, as has been reported by different labs working on SMA related issues. For this reason, the authors should report on motor neuron counts from vulnerable spinal segments (rostral lumbar segments where there is no clear delineation between LMC and MMC motor neurons). At bare minimum, the authors should report separately the number of motor neurons from LMC and MMC for figs 1h, 2f and 4d, since MMC motor neurons are vulnerable but LMC are resistant motor neurons in SMA mice.

3. The results for the analysis of the motor neuron soma area appears to be inaccurate in figs 1h, 2f and 4f. In all three graphs, the average soma area is shown to be approximately 5 square microns. This is simply wrong. Several previous studies have shown that adult a-motor neurons are in the range of 800-1000 square microns. I would therefore urge the authors to re-examine their calculations.

4. There is no information regarding neuromuscular junction (NMJ) innervation, since a hallmark of SMA is NMJ denervation. The authors are strongly advised to present data on NMJ innervation from vulnerable muscles. Several previous publications (PMID: 21968514; PMID: 28504671; PMID: 34825141) have reported on vulnerable and resistant muscles in SMA mice.

5. The evidence presented from Western blots are puzzling. First, it is unclear why the authors opt to use two different approaches to measure SMN expression (as in Fig.2b). In addition, why there is no SMN protein in controls at 12 months (fluorescent Western)? Furthermore, in Fig.2b, it appears that a fourth band exists for the "benchmark" but it is chopped. Yet, there are only 3 data points appearing in the graph for this group. Similarly, in Extended Data Fig.2, only two bands are shown for SMN for the liver, heart and quadriceps, but in the graphs, there are three data points! In the same figure, it appears that there are four bands for the "benchmark" treatment but the fourth band is cropped. The authors are advised to pay attention to detail and provide accurate representation of the raw data for the Western. Lastly, can the authors offer a rationale why they use two different loading controls in Fig.2b between 3 and 12 months (ie tubulin at 3 months and GAPDH at 12 months)?

6. The observation of SMN aggregation within the cytoplasm is striking as shown in Fig.2c, 2e, 4e and 4f. However, the authors assign these aggregates as Gemini of Cajal bodies or gems. I don't think this is the case. The Pellizzoni lab reported similar findings, however they demonstrated that SMN aggregates were SmB+ (SmB is a member of the Sm core that is assembled on small nuclear RNAs by SMN protein). Thus, the authors should examine whether these aggregates are SmB+ and in agreement with those previously reported (PMID: 33795885).

7. The issue of toxicity in DRG has not been properly addressed. Several previous reports raised the possibility that proprioceptive sensory neurons are involved in SMA pathogenesis. However, the authors use NeuN as a marker for sensory neurons which is not informative in the context of whether these sensory neurons are of proprioceptive origin or not. The authors should use antibodies that mark proprioceptive neurons (such as Parvalbumin or Runx3) and report the percentage of proprioceptive neurons exhibiting SMN aggregation. Is SMN aggregation in all sensory neurons or this is observed selectively? To this end, the authors present data in the Extended Figure 1c, where they show Vglut1 staining (in red) together with Hoeschst (in blue). The authors claim that there is no difference in Vglut1 in immunostaining in three different groups but they provide no quantification. Furthermore, Vglut1 staining in the ventral horn is not a marker of proprioceptive synapses, since cortico-spinal synapses are also Vglut1+ (PMID: 16786558; PMID: 22973013; PMID: 24997765). My understanding is that only Vglut1 synapses which make contact with the soma of motor neurons are of proprioceptive nature and for this reason, the authors should use Vglut1 and Chat immunostaining to quantify and report the proprioceptive synapses on motor neurons under the three experimental conditions (controls, "benchmark" and 2nd generation AAV).

8. In the authors' previous publication (PMID: 38413838, published EMBO Mol Med) the Ejection Fraction in the heart of control mice is different with that reported in this study (as shown in Extended Fig.3b). In their previous paper, the authors show that in Controls the Ejection Fraction is ~60% at 1 month old (Fig.4A in PMID:38413838) whereas in the current study, the Ejection Fraction in Controls is ~30% at 3 months old. If age is the reason, why there is no difference between 3 months and 10 months in Controls as shown in Extended Fig.3b? This issue is key, since the authors in the previous study show that at 1 month old, the ejection fraction in the heart from "benchmark"-treated mice was significantly reduced at 1 month old, but in the current study, event at 3 months old mice, there is no significant difference. Could the authors provide an explanation for this apparent discrepancy? In a similar argument, the stroke volume is also different between 1 month old control mice (previous paper PMID: 38413838) and 3 months old mice (the current study). This issue is of high relevance if the authors will argue towards cardiac defects due to "benchmark" AAV, especially since there is no difference between 3 and 10 months (as shown in the Extended Figure 3 in the current study) while their previous publication reports a large reduction following "benchmark" AAV (Fig.4A in PMID: 38413838). If this difference could be due to the administration route, then the authors should elaborate on the implications.

9. It is unclear how the authors can make a claim that their 2nd generation AAV provides improvement in motor behavior in SMA mice and yet, it is toxic in 20 month old control (healthy) mice. How do the authors reconcile with such opposing results?

Some minor issues that require attention:

1. The sentence (in lines 37-38) should include the provision that SMA is the leading genetic cause of infants, if left untreated.

2. The authors should provide the volume of the AAV injected ICV in all groups. They have provided the dose, but not the actual volume injected per animal.

3. The authors should provide the number of DRGs collected and used for Western blots.

4. The authors are advised to provide citations (papers and/or reviews) for their statement appearing in lines 44-45 (last sentence of the first para in the Introduction).

5. The authors sometimes refer to SMN1 and sometimes to SMN protein. Is there a difference? The authors should clarify.

6. In lines 260-262, where the authors discuss the role of SMN protein in pre-mRNA splicing, the authors should cite some

previous essential reviews by the Dreyfuss and Pellizzoni labs (ie PMID: 39694825; PMID: 15120993; PMID: 24769255; PMID: 17401408).

Reviewer #2

(Remarks to the Author)
The SMA field has witnessed many exciting developments over the past decade. Once a mostly deathly, progressive neuromuscular disease for most diagnosed newborns, the current outlook is very different. Three gene-targeting treatments are now available, and the realisation that very early treatment optimizes outcomes has led to the inclusion of SMA in many newborn screening programmes (NBS). Although long-term follow-up is still relatively limited, each of these gene-targeting treatments leads to significantly improved survival and clinical outcomes compared to the natural history of SMA.
One of the three gene-targeting treatments for SMA is onasemnogene abeparvovec (Zolgensma), an AAV9-based gene replacement therapy that is delivered IV and leads to systemic restoration of SMN expression by introducing an SMN1-containing episome. Because of its one-off nature, this is often the treatment of choice in babies diagnosed through NBS. However, very little is known about the long-term safety profile of systemic viral gene therapy, both in SMA and in general.
Previously, the authors of the current manuscript published a paper on their second-generation AAV9-based gene therapy for SMA. Their approach uses an endogenous, human SMN1 promotor rather than the hybrid CAG/CMV promotor used in Zolgensma. In the current manuscript, the authors present a long-term follow-up study on the development of their second-generation SMA gene therapy. They present a careful and extensive pathological of both treated SMA and wild-type mice that were treated at the day of birth with either a similar-to-Zolgensma benchmark vector or their second-generation vector. They show that their vector improves outcomes and survival compared to the benchmark vector. Importantly, the benchmark vector is shown to have carcinogenic properties in the long-term, by integrating into the mouse Rian locus in wildtype mice at 20 months. Additionally, SMA mice treated with the benchmark vector showed cardiac thrombosis at 12 months. Their second-generation vector, whilst improving these phenotypes, impaired motor function of wild-type mice after 20 months.
The comments I have are probably mostly more of a philosophical nature, in the sense that obviously if true in humans, this publication has the potential to cause significant unrest amongst the parents of the thousands of children that have already been treated with Zolgensma. For this reason, it will be important that the authors choose the words of the abstract, final introduction paragraph and ending of their discussion very carefully, perhaps including some disclaimers along the lines of the points mentioned below:

- Translatability from Mouse Models to Humans
The main issue here is the translational relevance from mice to humans. In general, the SMA field has been very lucky with the translatability of mouse models. Especially the observations from younger mice in severe SMA mouse models have proven to be reliable and highly translatable. It gets more challenging when mice get older. The development of reliable mouse models for other types of SMA, or more late-onset types of SMA, such as SMA type 3, hasn't really been very successful at all.
The current manuscript investigates the effect of treatments on older mice, but the extent to which mice can be used at all to study aging-related situations or phenotypes is open for discussion. Here, both the cardiac phenotypes in 12-month-old SMA mice and the liver cancer in 20-month-old wild-type mice have potential big implications in humans. To reliably prove such events, one would need to most likely move to primates at some point or possibly organoids. The challenge with organoids and primates will be how can we compare certain time points in vitro or in vivo to timelines in humans. That's going to be very challenging and it's very relevant, and the crude reality is probably that time will tell what will happen in the wider, Zolgensma-treated patient population.

- Delivery Route: IV vs ICV Injection
For the current manuscript, I would like to know a little bit more about the choice of moving from IV to ICV injection compared to the previous paper, because I think that that's not something that the authors have explained very clearly. This is important because the elevated liver toxicity profile for the benchmark vector is solved by switching to ICV instead of IV injection. Still, in mice, ICV injection at P0 probably also leads to systemic treatment, less so than in IV-treated mice, but I think it's important to discuss this in more detail because patients are treated mostly IV, even though there are trials underway to test ICV treatment with Zolgensma. So, IV injection is probably most relevant for patients. That is what the authors described in their previous EMBO Mol Med publication, but not in the current manuscript, and so I would be very interested to hear in detail the rationale behind this choice.

- Comparison with Previous Publications
In line with this, the comparison between the current paper and the authors' previous paper warrants further discussion. As far as I can tell, the timing and the dosage of the treatment is comparable. The delivery route isn't. And then, obviously, the rest of the publication is aimed at different ages and time points. The fact that the delivery routes are different makes it also more difficult to make direct comparisons between the two publications.
Another relevant paper was published recently by a group that used a similar approach to optimize gene therapy by changing the promoter. This paper by Song et al. came out recently in Molecular Therapy Methods & Clinical Development, and I think it would be nice if the authors could discuss this paper. This manuscript uses a hybrid CMV with a neuronal promoter sequence and addressed the issue of normalizing SMN levels in the long term, especially in peripheral tissues.

- Mechanisms of AAV Toxicity
What I'm still not convinced about is that we have this conundrum of AAV treatment leading to toxicity. Often, the idea with AAV treatment and especially liver toxicity is that it's mostly due to very high level AAV exposure leading to immune system

activation. This is why we're treating children with corticosteroids, et cetera, when they start Zolgensma treatment. However, this paper and also the other promoter-optimizing paper by Song et al. both suggest that the primary driver of toxicity is SMN overexpression rather than AAV exposure, or that at least it plays an important role. Clearly the liver carcinoma part is more due to the AAV integration, so it's a separate mechanism of toxicity that we must deal when using these kinds of therapies. In summary it's a complex situation where the conclusion might be that different time points in development and in exposure to the therapy require different ways of managing the treatment. For example, as the authors point out, the fact that DRG toxicity might be mitigated by the addition of a miRNA sequence in the viral vector could be one approach, but clearly the risk of AAV integration in the genome or the regulation of SMN expression over time are separate safety challenges that would require a different approaches to mitigate.

- Additional Point Requiring Clarification
I would be grateful if the authors could add more detail on what they mentioned about the difference between the mouse Rian locus, which apparently is prone to AAV integration, but does not occur in other species. The authors refer to one specific reference about 1-3% rAAV integration in hepatocytes but I would like to see some more detail on this point and how likely it could potentially be for patients in the discussion.

Reviewer #3

(Remarks to the Author)
This is an interesting manuscript that reports the longterm outcomes of SMA and WT mice dosed ICV with various doses of two forms of AAV9-SMN gene therapy. This group has previously published a detailed study describing a comparison of shorter term outcomes after dosing with a "benchmark" vector, a zolgensma like vector in which a SMN1 cDNA is driven by the chicken beta actin (CBA) promoter and a CMV enhancer versus a 2nd generation vector they designed, in which a SMN1 transgene is driven by a fragment of the SMN1 promoter. In their prior study mice were studied out to approximately 90 days (Xie Q et al EMBO Mol Med 204). In this study, their 2nd generation vector showed improved SMA disease outcomes and safety. Because there are ongoing concerns about longterm toxicities of AAV9 gene therapies, the group is now assessing longterm outcomes following various doses of these viral vectors delivered ICV.

-With 1.1E+13 vg/kg, both treated groups were tracked out to 75 days (seemingly very similar to previously published study). The 2nd generation vector showed enhanced SMN protein expression, improved survival and modest improvements in motor behavior, but equal number of motor neurons.
-At a higher viral dose of 3.3E+13 vg/kg, mice were tracked to 360 days. There was again better induction of SMN protein levels, enhanced survival, modest improvements in motor behavior, but no change in motor neuron number with the second generation vector. At 360 days, more cardiac thrombosis was detected in mice treated with the benchmark vector (3 of 7) compared to the second generation vector (0 of 8). This was associated with more SMN expression in the left atrium in the benchmark vector mice. When a codon optimized version of the vector was used driving even higher expression, the rate of cardiac thrombosis worsened.
-Finally a dose of 1.0E+14 vg/kg was used only in WT mice and they were tracked to 20 months. Again SMN protein expression and survival were better in the second generation vector treated mice, BUT at 20 months behavior worsened and SMN aggregates were seen in MNs and DRGs. Such aggregates and toxicity associated with overexpression of SMN was previously described by Van Alstyne et al. Nat Neurosci 2021. In this group of mice, the authors also see higher rates of hepatocellular carcinoma in the benchmark vector treated mice due to integrations at the Rian locus-a phenomena that is well described to be specific to mice (this locus is not present in other species).
Based on the data the conclusion of the authors as stated in the last sentence of the abstract is that the results raise caution about the longterm efficacy and safety of treating patients with zolgensma.

Concerns:
1) Although the longterm safety of AAV based gene therapies is of high importance and broad interest, I am not convinced that this manuscript as presented adds substantial new insights that inform "the longterm efficacy and safety of treating patients with zolgensma." It has already been reported that ICV-delivery of a AAV9-SMN vector driven by the GUSB promoter can result in aggregation of SMN protein in neurons and neurodegeneration (Van Alstyne et al.). It has also been previously demonstrated that AAV vectors at high dose in mice cause HCC in a species-specific way and this locus-specific integration is not relevant to humans. The only novel toxicity is the cardiac thrombosis. Also regarding human relevance, it is not clear that the findings observed with ICV injection of AAV9-AAV are relevant to the current FDA approved zolgensma which is delivered iv. The results of IT delivery of zolgensma have been presented in abstract form: the relative dose of virus used in the human studies is significantly less than the doses being used in humans.
2) Regarding the comparisons of SMA disease efficacy with the different vectors, this manuscript does demonstrate that the improved efficacy with the second generation vector persists at later time points, but this seems to be an incremental advance compared to their thorough, previously published work.
3) The authors should justify the virus doses used particularly when trying to make conclusions regarding Zolgensma dosing in humans. The doses of zolgensma used in the IT trials in humans are significantly lower than those used in the mice (see STRONG study). The highest dose of virus was only tested in WT mice-was this because it was not tolerated in SMA mice?
4) THe authors need to define and quantify aggregates of SMN not just show single images. Are these aggregates associated with neurodegeneration as previously reported?
5) There is no histological evidence that the second generation vector outperforms the benchmark. MN numbers are equivalent amongst experimental groups. Electrophysiology, NMJ or myofiber pathological assessment would help provide

convincing evidence that one vector outperforms another in terms of the most clinical relevant cell types: motor neurons and myofibers.
6) The authors should be more upfront about the hepatocellular carcinoma being species specific in the abstract. Although discussed in the discussion, it needs to mentioned earlier.
7) The authors make reference to results of the IT dosing Zolgensma clinical trials. The authors should review the abstracts reporting these results from the World Muscle Society meeting and Cure SMA meetings that have been posted on the Novartis website. The efficacy is extremely modest. THis should be more accurately reported in the introduction.

Version 1:

Reviewer comments:

Reviewer #1

(Remarks to the Author)
The authors have performed additional experiments and revised their manuscript accordingly.
I have no new concerns.

Reviewer #2

(Remarks to the Author)
it would have been helpful to get a more detailed reply than 'see line XX' but I think I went through everything and with the reponse to the other reviewers belief all important points have been addressed sufficiently

Reviewer #3

(Remarks to the Author)
The authors have done a very good job of addressing the concerns that were raised during the initial review. They have edited the manuscript to make clear that HCC may be a rodent specific phenomena. In addition, the authors have performed new experiments to quantify motor neuron number and size, NMJ innervation and determine CMAPs. In addition SMN aggregates have been quantified in DRGs.

I have a few remaining concerns:
1) For this reviewer, normalizing motor neuron number and size values to 100% is not appropriate and makes it very difficult to judge the validity of these assessments. I strongly recommend showing absolute values for MN number and MN size. In addition for most of these analyses, there do not appear to be statistically significant differences observed between the benchmark vector and the 2ndGen vector. Given this, these results should not be overstated (a lack of difference with WT and 2nd gen mice is less convincing than a statistically significant improvement between 2nd generation vs. benchmark vector-treated mice).
2) Both reviewer 1 (point#9) and reviewer 3 (point #3) raised concerns about claims that 2nd gen vector is safer, BUT WT mice treated with a high dose showed more motor behavioral abnormalites than bench mark vector (figure 4). The authors speculate that this may be due to presence of mouse Smn in addition to virally-delivered SMN. They note in response letter that during the revision period they treated SMA mice with high dose-I think the longterm results of the effects of the bench mark vs. 2nd gen high dose vector in the SMA mice would be ideal to include.
3) The publications reporting the experience with IT zolgensma in humans have now been published (PMID: 41360995 and PMID: 41360993). These manuscripts need to be cited and appropriately discussed. I remain concerned about the wording of the last phrase of the abstract: "...these findings raise concerns regarding the long-term safety and efficacy of Zolgensma in SMA patients." Given the many differences between mice and humans (anatomy, CSF volume, blood brain barrier, immunology, etc), I think this conclusion requires softening. On this theme, a point that has not been sufficiently addressed in the discussion is the relative doses of virus per CSF volume administered in mice in this study vs. human clinical use. In humans, 1.1x10(14)vg is given IT with a human CSF volume estimated at 100-150 ml. In contrast, mouse CSF volume is ~35-50 ul. I think some discussion of the estimated concentrations of virus in CSF is important to include particularly as level of SMN overexpression seems to be a key determinant of toxicity.

Version 2:

Reviewer comments:

Reviewer #3

(Remarks to the Author)
I very appreciate that the authors thoroughly addressed the remaining concerns.

Open Access This Peer Review File is licensed under a Creative Commons Attribution 4.0 International License, which permits use, sharing, adaptation, distribution and reproduction in any medium or format, as long as you give appropriate credit to the original author(s) and the source, provide a link to the Creative Commons license, and indicate if changes were made.
In cases where reviewers are anonymous, credit should be given to 'Anonymous Referee' and the source.
The images or other third party material in this Peer Review File are included in the article's Creative Commons license, unless indicated otherwise in a credit line to the material. If material is not included in the article's Creative Commons license and your intended use is not permitted by statutory regulation or exceeds the permitted use, you will need to obtain permission directly from the copyright holder.
To view a copy of this license, visit https://creativecommons.org/licenses/by/4.0/

Manuscript NCOMMS-25-46620

Response to Reviewers

Reviewers' Comments to the Authors:

Reviewer #1 (Remarks to the Author):
The manuscript entitled "Long-Term Comparative Analysis of AAV9-Mediated Gene-Replacement Therapies for Spinal Muscular Atrophy in Mice" by Chen and colleagues report on long-term severe side effects in pre-clinical models following a genetic delivery (Zolgensma-LIKE AAV) of the SMN gene that has been FDA approved in severe SMA patients. In addition, they use SMA mice to compare the effects of a second generation vector which appears to provide no side effects and better outcome in mutant mice. The severe side effects of the Zolgensma vector (assigned as "benchmark" vector in the study) include cardiac defects (thrombosis) but more alarmingly, tumors in liver and lungs in SMA mice after several months post-administration. This study is important and timely due to the ongoing therapy of SMA patients, particularly the severe form of Type 1 SMA patients. Intriguingly, the authors report on the benefits in SMA mice following the administration of a novel vector expressing a codon-optimized hSMN1 transgene driven by the endogenous hSMN1 promoter. They report no adverse effects of the tumorigenesis observed under the Zolgensma-like vector, which also shows a more robust benefit in motor behavior which was dose-dependent. However, administration of this vector in healthy (or wild type control) mice resulted in sensory toxicity after twenty months from its administration. The study provides important insights and sheds light on the long-term potential toxicity of Zolgensma in SMA patients. The second-generation AAV is not novel since the same authors reported its benefits in 2024 (EMBO Mol Med). The novelty of the current study regarding this AAV is the route of administration; being intracerebroventricular (or ICV) compared to intravenous (or IV) reported previously in 2024. The study is well designed and for most of the experiments well executed with reasonable interpretations. However, several aspects of the study require additional attention to detail, further experimentation and new analysis since there are some serious concerns regarding the validity of their conclusions.

We thank the reviewer for the positive comments and appreciate the suggestions to improve the quantity of our research.

The major concerns are explained below:

1. There is a general problem with reporting statistical results. I urge the authors to pay particular attention to detail for all results. I amplify my concerns by raising the following issues:
i) in Fig.1d, the authors report the p values, but they don't mention which groups they compare; is it between the "benchmark" and 2nd generation AAV or is between 2nd generation and SMA mice?

We thank the reviewer for pointing out this issue. We have now carefully labeled the comparison and indicated the statistical methods in all the figures. For Fig. 1d, the comparison is between the benchmark and the 2nd-generation vector.

ii) The comparison in Fig.1e is not valid because the authors compare the total number of observations, rather than the average number of observations per mouse. By doing so, the authors compare a high number of data points, but at the same time this is considered to be biased since due to pseudo replication. The authors should compare the average value per each animal across the different groups. In addition, the authors state in the figure legend that this comparison was performed using one way ANOVA with Dunnetts' post hoc testing; however, they only report p value between "benchmark" and 2nd generation AAV. The authors are encouraged to report p values between controls (black data points) and the two AAV approaches. In this manner, the reader will be

able to get a better understanding of how strong the latency of the two experimental groups were to the values from the control mice.

We are sorry for the confusion. In Fig. 1e, each data point represents an individual mouse. We have clarified this in the figure legend. We also reported the p-values comparing the healthy controls with the two AAV approaches, as suggested.

iii) In Fig.2, the authors compare the results between "benchmark" and the higher dose (3.3E+ 13vg/kg) of the 2nd generation AAV. However, this comparison is not informative nor valid. The authors should compare the results between the doses (the low one, ie 1.1E+13vg/kg and the higher one, ie 3.3E+ 13vg/kg). Without such statistical comparison the authors cannot conclude that the higher dose is better than the lower dose. This comparison should include % survival, righting reflex, latency to fall, descent latency (inverted grid) for at least one (but preferably two) time point(s).

We agree with the reviewer. Now we have analyzed the dose responses from AAV treatment, and the data were summarized as Supplementary Fig. 2

iv) In fig.2d, the p value presented in the graph is confusing. It shows that comparison between HC and 2nd generation is p=0.5 (i.e. not significant) where comparison between HC and "benchmark" the p value is <0.001, which judging from the graph, this is simply not the case. The authors should recheck the analysis.

The P values in Fig. 2d were swapped. We have now made corrections (see now Supplementary Fig. 4) and updated the band colors (red for SMN and green for Gapdh) to be consistent throughout the manuscript.

v) The authors need to report the number of mice per experimental group used, rather than the number of data points; for instance, the motor neuron counts presented in three different instances, is unclear how many mice have been used (in figs 1h, 2f and 4d).

We used 4-5 mice for quantification and have included this information in the corresponding figure legends.

2. The method for assessing motor neuron survival is flawed. First, in Figs 1h, 2f and 4d, it is unclear whether the authors have included both LMC and MMC motor neurons. Irrespective, LMC motor neurons are considered resistant motor neurons (i.e. do not degenerate) under SMN deficiency conditions, as has been reported by different labs working on SMA related issues. For this reason, the authors should report on motor neuron counts from vulnerable spinal segments (rostral lumbar segments where there is no clear delineation between LMC and MMC motor neurons). At bare minimum, the authors should report separately the number of motor neurons from LMC and MMC for figs 1h, 2f and 4d, since MMC motor neurons are vulnerable, but LMC are resistant motor neurons in SMA mice.

We appreciate the reviewer for pointing this out. Now we quantified the MNs in LMC and MMC separately from the L5 and analyzed the MNs in L1. The new data are shown in Figs 1h, 2e, 4e; Supplementary Figures 1, 5, and 10.

3. The results for the analysis of the motor neuron soma area appear to be inaccurate in figs 1h, 2f and 4f. In all three graphs, the average soma area is shown to be approximately 5 square microns. This is simply wrong. Several previous studies have shown that adult a-motor neurons are in the range of 800-1000 square microns. I would therefore urge the authors to re-examine their calculations.

We apologize for the errors. The soma sizes in figs 1h, 2f and 4f should be 5 x 100 square microns. We labeled the x100 at the top of the y-axis in Figs 2f and 4f, but missed it in Fig. 1h. To simplify the comparison, we now use the HC group to normalize the changes as others did (PMID: 33795885). We have updated the Figs 1h, 2e, and 4e; Supplementary Figures 1, 5, and 10.

4. There is no information regarding neuromuscular junction (NMJ) innervation, since a hallmark of SMA is NMJ denervation. The authors are strongly advised to present data on NMJ innervation from vulnerable muscles. Several previous publications (PMID: 21968514; PMID: 28504671; PMID: 34825141) have reported vulnerable and resistant muscles in SMA mice.

We thank the reviewer for the suggestion. Quadratus lumborum (QL) muscles are particularly vulnerable in the SMA mice. We conducted immunostaining with Neurofilament-M (NF) to visualize presynaptic axons, using Synaptophysin (SYP) to detect presynaptic motor nerve terminals, and α-bungarotoxin (BTX) to stain post-synaptic acetylcholine receptors in the QL muscles of SMA. The new data is shown as Fig.1j.

5. The evidence presented from Western blots are puzzling. First, it is unclear why the authors opt to use two different approaches to measure SMN expression (as in Fig.2b). In addition, why there is no SMN protein in controls at 12 months (fluorescent Western)? Furthermore, in Fig.2b, it appears that a fourth band exists for the "benchmark" but it is chopped. Yet, there are only 3 data points appearing in the graph for this group. Similarly, in Extended Data Fig.2, only two bands are shown for SMN for the liver, heart and quadriceps, but in the graphs, there are three data points! In the same figure, it appears that there are four bands for the "benchmark" treatment, but the fourth band is cropped. The authors are advised to pay attention to detail and provide accurate representation of the raw data for the Western. Lastly, can the authors offer a rationale why they use two different loading controls in Fig.2b between 3 and 12 months (ie tubulin at 3 months and GAPDH at 12 months)?

We thank the reviewer for pointing out these issues.

1. In Fig. 2b, the Western blot was imaged in two different scanners, resulting in inconsistency. The SMN signal in controls at 12 months was faint due to low exposure because we tried to avoid overexposure of the SMN signal. The fourth band in the BMK group is similar to the other three mice in the same group, so we did not include it in the image. Now we have conducted the WB again for these samples to make them consistent and updated Fig. 2b.
2. Sorry for the confusion in Extended Fig. 2 (now Supplementary Fig. 3). We only included two mice in the untreated SMA group and did not include them in the quantification. Comparison was performed in the other three groups (n = 3 each in the WT, 2nd-G, and BMK groups).
3. Sorry for the confusion that Tubulin and Gapdh were used for loading control. Previously, we used Tubulin as a loading control in our previous publication (EMBO Molecular Medicine PMID: 38413838, 2024). At that time, one reviewer raised a concern that the absence of the SMN protein may affect tubulin levels, leading to inaccurate SMN protein quantification. We compared SMN protein quantification using Tubulin or Gapdh as loading controls and found no significant difference. After that, we switched the loading control to Gapdh, but some WB analyses using Tubulin in this study were done at that time. Now, we have re-run the WB analysis using Gapdh instead of Tubulin as the loading control in this study. The new WB data were shown as Fig. 2b.

6. The observation of SMN aggregation within the cytoplasm is striking as shown in Fig.2c, 2e, 4e and 4f. However, the authors assign these aggregates as Gemini of Cajal bodies or gems. I don't think this is the case. The Pellizzoni lab reported similar findings, however they demonstrated that SMN aggregates were SmB+ (SmB is a member of the Sm core that is assembled on small nuclear

RNAs by SMN protein). Thus, the authors should examine whether these aggregates are SmB+ and in agreement with those previously reported (PMID: 33795885).

We thank the reviewer for the suggestion. To examine these aggregates as SmB+, we performed co-staining for SMN and SmB in ChAT(+) and PV(+) neurons. We observed that overexpression of SMN led to cytoplasmic aggregations of SmB in both L5 motor neurons and PV neurons in L5 DRGs, a finding similar to that reported by the Pellizzoni lab. These data were shown in the updated Figs. 2c, 2d, 4f, and 4g.

7. The issue of toxicity in DRG has not been properly addressed. Several previous reports raised the possibility that proprioceptive sensory neurons are involved in SMA pathogenesis. However, the authors use NeuN as a marker for sensory neurons which is not informative in the context of whether these sensory neurons are of proprioceptive origin or not. The authors should use antibodies that mark proprioceptive neurons (such as Parvalbumin or Runx3) and report the percentage of proprioceptive neurons exhibiting SMN aggregation. Is SMN aggregation in all sensory neurons or this is observed selectively? To this end, the authors present data in the Extended Figure 1c, where they show Vglut1 staining (in red) together with Hoeschst (in blue). The authors claim that there is no difference in Vglut1 in immunostaining in three different groups but they provide no quantification. Furthermore, Vglut1 staining in the ventral horn is not a marker of proprioceptive synapses, since cortico-spinal synapses are also Vglut1+ (PMID: 16786558; PMID: 22973013; PMID: 24997765). My understanding is that only Vglut1 synapses which make contact with the soma of motor neurons are of proprioceptive nature and for this reason, the authors should use Vglut1 and Chat immunostaining to quantify and report the proprioceptive synapses on motor neurons under the three experimental conditions (controls, “benchmark” and 2nd generation AAV).

We thank the reviewer for this critical point. We performed the following experiments:

1) We co-stained PV and SMN in the DRGs of the SMA mice that received AAV vectors at 3.3E+13 vg/kg for three months. Age-matched healthy mice were used for comparison. We observed that SMN aggregates are present in ~50% and ~80% of PV+ proprioceptive neurons (PNs) in the BMK and 2nd-G groups, respectively. The new data is included in Fig. 2d.
2) We also co-stained PV and SMN in the DRGs of the C57BL/6 mice that received AAV vectors at 1.0E+14 vg/kg or PBS for 20 months. We observed that SMN aggregates are present in ~10% and ~60% of PV+ proprioceptive neurons (PNs) in the BMK group and 2nd-G group, respectively. The new data is included in Fig 4f and Supplementary Fig. 10.
3) We performed co-staining for Vglut1 and ChAT to quantify the proprioceptive synapses on motor neurons. The new data is shown as Supplementary Fig. 6.

8. In the authors’ previous publication (PMID: 38413838, published EMBO Mol Med) the Ejection Fraction in the heart of control mice is different with that reported in this study (as shown in Extended Fig.3b). In their previous paper, the authors show that in Controls the Ejection Fraction is ~60% at 1 month old (Fig.4A in PMID:38413838) whereas in the current study, the Ejection Fraction in Controls is ~30% at 3 months old. If age is the reason, why is there no difference between 3 months and 10 months in Controls as shown in Extended Fig.3b? This issue is key, since the authors in the previous study show that at 1 month old, the ejection fraction in the heart from “benchmark”-treated mice was significantly reduced at 1 month old, but in the current study, event at 3 months old mice, there is no significant difference. Could the authors provide an explanation for this apparent discrepancy? In a similar argument, the stroke volume is also different between 1 month old control mice (previous paper PMID: 38413838) and 3 months old mice (the current study). This issue is of high relevance if the authors will argue towards cardiac defects due to “benchmark” AAV, especially since there is no difference between 3 and 10 months (as shown in the Extended Figure 3 in the current study) while their previous publication reports a large reduction following “benchmark” AAV (Fig.4A in PMID:

38413838). If this difference could be due to the administration route, then the authors should elaborate on the implications.

Measurement of in vivo mouse cardiac function is prone to a large degree of variability. Heart function can vary with age, gender, stress, heart rate, blood pressure, and many other factors (PMID: 29351456). Furthermore, although isoflurane is the most common anesthetic used, it is a cardio depressant and can reduce systolic function. We try to control these factors by using animals of the same age, imaging at similar heart rates (all above 450 bpm), and using the same dose of isoflurane (2.5% 1 l/min). In our first paper, the IV-delivered benchmark vector showed declines in EF, FS, and stroke volume compared with healthy controls and the second-generation virus at one month. We hypothesize this reduction was due to frequent premature ventricular contractions which can reduce cardiac function. Ejection fraction is a measurement of cardiac function, and fractional shortening and stroke volume are directly correlated. In the present paper, the measured ejection fraction of healthy control mice at 3 and 10 months is lower than that observed in our first study. There are several factors that could be responsible for this difference. Blood pressure may be higher in mice at 3 and 10 months of age; this can reduce cardiac function. Unfortunately, we could not directly measure blood pressure. Second, it is possible animals were under greater stress in the present study. Many other factors could have caused a reduced ejection fraction in healthy control mice in the present study. We did not conclude that the benchmark caused reduced cardiac function; however, we observed increased cardiac thrombosis in the benchmark-treated animals. The stroke volumes are lower in the present study, because stroke volume is directly correlated with ejection fraction, which is uniformly lower in the present study. The ECHO test was conducted by the Cardiovascular Physiology Core at Umass. The same cohort of mice (1-, 3-, or 10-month-old) was analyzed side-by-side to ensure a consistent comparison among these three groups (HC, BMK- and 2nd-G vector-treated SMA mice). In the first study, premature ventricular contractions were observed in the benchmark-treated group, and we did not observe that pathology in the present study. In our first paper, the IV-delivered benchmark vector generated 69-fold more SMN protein than in WT mice after one month of treatment. However, the benchmark vector treatment increased the SMN protein level by about one-fold after switching to ICV injection in this study (Supplementary Fig. 3a, new data). Possibly the supraphysiological expression of SMN (69-fold high) led to premature ventricular contractions rapidly, but a two-fold high SMN is tolerable in the early stage. We also discussed the implications of administration routes in the manuscript (lines 64-65; 189-193).

9. It is unclear how the authors can make a claim that their 2nd generation AAV provides improvement in motor behavior in SMA mice and yet, it is toxic in 20-month-old control (healthy) mice. How do the authors reconcile with such opposing results?

The discrepancy between SMA and WT mice may result from differences in SMN expression level and duration. Here are the differences between the studies on SMA and WT mice.

1) The dose in WT and SMA mice is 1.0E+14 and 1.0-3.3E+13 vg/kg, respectively.
2) The treatment in WT and SMA mice is 20 and 12 months, respectively.
3) The WT but not the SMA mice express endogenous Smn protein

Although we did not observe toxicity in SMA mice, it may occur in a >12-month study at a higher dose. In our first paper, we demonstrated that the supraphysiological expression of SMN in the liver and heart from the benchmark vector is harmful. In this paper, we again showed that high-level SMN expressions are associated with cardiac thrombosis. Overall, AAV-mediated gene transfer in the CNS is still limited. The benchmark vector could not express sufficient SMN protein for therapeutic purposes in the CNS of SMA mice, but it was tolerated in WT mice. In contrast, the 2nd-G vector can express adequate SMN for therapeutic use but can cause detrimental neurological effects if the expression level is too high. We are currently working on a next-generation vector to make the SMN

expression within the CNS safe and effective. We also discussed the differences in motor function improvement from these two vectors in the Discussion (lines 326-333).

Some minor issues that require attention:
1. The sentence (in lines 37-38) should include the provision that SMA is the leading genetic cause of infants, if left untreated.

We have included this in the manuscript (lines 39-40).

2. The authors should provide the volume of the AAV injected ICV in all groups. They have provided the dose, but not the actual volume injected per animal.

We injected 4 μL into one mouse and 2 μL into each hemisphere. We have included it in the Methods (line 420).

3. The authors should provide the number of DRGs collected and used for Western blots.

We collected as many DRGs as possible from each mouse (46-48 per mouse) for WB analysis. We have now included this information in the legend (Supplementary Fig. 4).

4. The authors are advised to provide citations (papers and/or reviews) for their statement appearing in lines 44-45 (last sentence of the first para in the Introduction).

The citations have now been included.

5. The authors sometimes refer to SMN1 and sometimes to SMN protein. Is there a difference? The authors should clarify.

SMN1 and SMN are referred to as gene and protein, respectively. We have now revised the manuscript accordingly.

6. In lines 260-262, where the authors discuss the role of SMN protein in pre-mRNA splicing, the authors should cite some previous essential reviews by the Dreyfuss and Pellizzoni labs (ie PMID: 39694825; PMID: 15120993; PMID: 24769255; PMID: 17401408).

We have cited these papers as suggested.

Reviewer #2 (Remarks to the Author):

The SMA field has witnessed many exciting developments over the past decade. Once a mostly deathly, progressive neuromuscular disease for most diagnosed newborns, the current outlook is very different. Three gene-targeting treatments are now available, and the realisation that very early treatment optimizes outcomes has led to the inclusion of SMA in many newborn screening programmes (NBS). Although long-term follow-up is still relatively limited, each of these gene-targeting treatments leads to significantly improved survival and clinical outcomes compared to the natural history of SMA.
One of the three gene-targeting treatments for SMA is onasemnogene abeparvovec (Zolgensma), an AAV9-based gene replacement therapy that is delivered IV and leads to systemic restoration of SMN expression by introducing an SMN1-containing episome. Because of its one-off nature, this is often the treatment of choice in babies diagnosed through NBS. However, very little is known about the long-term safety profile of systemic viral gene therapy, both in SMA and in general. Previously, the

authors of the current manuscript published a paper on their second-generation AAV9-based gene therapy for SMA. Their approach uses an endogenous, human SMN1 promotor rather than the hybrid CAG/CMV promotor used in Zolgensma. In the current manuscript, the authors present a long-term follow-up study on the development of their second-generation SMA gene therapy. They present a careful and extensive pathological of both treated SMA and wild-type mice that were treated at the day of birth with either a similar-to-Zolgensma benchmark vector or their second-generation vector. They show that their vector improves outcomes and survival compared to the benchmark vector. Importantly, the benchmark vector is shown to have carcinogenic properties in the long-term, by integrating into the mouse Rian locus in wildtype mice at 20 months. Additionally, SMA mice treated with the benchmark vector showed cardiac thrombosis at 12 months. Their second-generation vector, whilst improving these phenotypes, impaired motor function of wild-type mice after 20 months.
The comments I have are probably mostly more of a philosophical nature, in the sense that obviously if true in humans, this publication has the potential to cause significant unrest amongst the parents of the thousands of children that have already been treated with Zolgensma. For this reason, it will be important that the authors choose the words of the abstract, final introduction paragraph and ending of their discussion very carefully, perhaps including some disclaimers along the lines of the points mentioned below:

We do agree with the reviewer that a cautious statement is essential. We have revised the manuscript according to the suggestions from the reviewer (lines 32-33; 73-76; 97-99; 368-372; 390-393).

- Translatability from Mouse Models to Humans
The main issue here is the translational relevance from mice to humans. In general, the SMA field has been very lucky with the translatability of mouse models. Especially the observations from younger mice in severe SMA mouse models have proven to be reliable and highly translatable. It gets more challenging when mice get older. The development of reliable mouse models for other types of SMA, or more late-onset types of SMA, such as SMA type 3, hasn't really been very successful at all.
The current manuscript investigates the effect of treatments on older mice, but the extent to which mice can be used at all to study aging-related situations or phenotypes is open for discussion. Here, both the cardiac phenotypes in 12-month-old SMA mice and the liver cancer in 20-month-old wild-type mice have potential big implications in humans. To reliably prove such events, one would need to most likely move to primates at some point or possibly organoids. The challenge with organoids and primates will be how can we compare certain time points in vitro or in vivo to timelines in humans. That's going to be very challenging and it's very relevant, and the crude reality is probably that time will tell what will happen in the wider, Zolgensma-treated patient population.

Please see lines 380-393

- Delivery Route: IV vs ICV Injection
For the current manuscript, I would like to know a little bit more about the choice of moving from IV to ICV injection compared to the previous paper, because I think that that's not something that the authors have explained very clearly. This is important because the elevated liver toxicity profile for the benchmark vector is solved by switching to ICV instead of IV injection. Still, in mice, ICV injection at P0 probably also leads to systemic treatment, less so than in IV-treated mice, but I think it's important to discuss this in more detail because patients are treated mostly IV, even though there are trials underway to test ICV treatment with Zolgensma. So, IV injection is probably most relevant for patients. That is what the authors described in their previous EMBO Mol Med publication, but not in the current manuscript, and so I would be very interested to hear in detail the rationale behind this choice.

Please see lines 64-65; 190-194; 246-247; 311-314

- Comparison with Previous Publications
In line with this, the comparison between the current paper and the authors' previous paper warrants further discussion. As far as I can tell, the timing and the dosage of the treatment is comparable. The delivery route isn't. And then, obviously, the rest of the publication is aimed at different ages and time points. The fact that the delivery routes are different makes it also more difficult to make direct comparisons between the two publications.
Another relevant paper was published recently by a group that used a similar approach to optimize gene therapy by changing the promoter. This paper by Song et al. came out recently in Molecular Therapy Methods & Clinical Development, and I think it would be nice if the authors could discuss this paper. This manuscript uses a hybrid CMV with a neuronal promoter sequence and addressed the issue of normalizing SMN levels in the long term, especially in peripheral tissues.

Please see lines 64-67; 190-194; 246-247; 300-304

- Mechanisms of AAV Toxicity
What I'm still not convinced about is that we have this conundrum of AAV treatment leading to toxicity. Often, the idea with AAV treatment and especially liver toxicity is that it's mostly due to very high level AAV exposure leading to immune system activation. This is why we're treating children with corticosteroids, et cetera, when they start Zolgensma treatment. However, this paper and also the other promoter-optimizing paper by Song et al. both suggest that the primary driver of toxicity is SMN overexpression rather than AAV exposure, or that at least it plays an important role. Clearly the liver carcinoma part is more due to the AAV integration, so it's a separate mechanism of toxicity that we must deal when using these kinds of therapies.
In summary it's a complex situation where the conclusion might be that different time points in development and in exposure to the therapy require different ways of managing the treatment. For example, as the authors point out, the fact that DRG toxicity might be mitigated by the addition of a miRNA sequence in the viral vector could be one approach, but clearly the risk of AAV integration in the genome or the regulation of SMN expression over time are separate safety challenges that would require a different approaches to mitigate.

Please see lines 300-310; 375-378

- Additional Point Requiring Clarification
I would be grateful if the authors could add more detail on what they mentioned about the difference between the mouse Rian locus, which apparently is prone to AAV integration, but does not occur in other species. The authors refer to one specific reference about 1-3% rAAV integration in hepatocytes but I would like to see some more detail on this point and how likely it could potentially be for patients in the discussion.

Please see lines 364-368

Reviewer #3 (Remarks to the Author):

This is an interesting manuscript that reports the longterm outcomes of SMA and WT mice dosed ICV with various doses of two forms of AAV9-SMN gene therapy. This group has previously published a detailed study describing a comparison of shorter term outcomes after dosing with a "benchmark" vector, a zolgensma like vector in which a SMN1 cDNA is driven by the chicken beta actin (CBA) promoter and a CMV enhancer versus a 2nd generation vector they designed, in which a SMN1 transgene is driven by a fragment of the SMN1 promoter. In their prior study mice were studied out to approximately 90 days (Xie Q et al EMBO Mol Med 204). In this study, their 2nd generation vector showed improved SMA disease outcomes and safety. Because there are ongoing concerns about

longterm toxicities of AAV9 gene therapies, the group is now assessing longterm outcomes following various doses of these viral vectors delivered ICV.
-With 1.1E+13 vg/kg, both treated groups were tracked out to 75 days (seemingly very similar to previously published study). The 2nd generation vector showed enhanced SMN protein expression, improved survival and modest improvements in motor behavior, but equal number of motor neurons.
-At a higher viral dose of 3.3E+13 vg/kg, mice were tracked to 360 days. There was again better induction of SMN protein levels, enhanced survival, modest improvements in motor behavior, but no change in motor neuron number with the second generation vector. At 360 days, more cardiac thrombosis was detected in mice treated with the benchmark vector (3 of 7) compared to the second generation vector (0 of 8). This was associated with more SMN expression in the left atrium in the benchmark vector mice. When a codon optimized version of the vector was used driving even higher expression, the rate of cardiac thrombosis worsened.
-Finally a dose of 1.0E+14 vg/kg was used only in WT mice and they were tracked to 20 months. Again SMN protein expression and survival were better in the second generation vector treated mice, BUT at 20 months behavior worsened and SMN aggregates were seen in MNs and DRGs. Such aggregates and toxicity associated with overexpression of SMN was previously described by Van Alstyne et al. Nat Neurosci 2021. In this group of mice, the authors also see higher rates of hepatocellular carcinoma in the benchmark vector treated mice due to integrations at the Rian locus-a phenomena that is well described to be specific to mice (this locus is not present in other species). Based on the data, the conclusion of the authors, as stated in the last sentence of the abstract, is that the results raise caution about the long-term efficacy and safety of treating patients with Zolgensma.

Concerns:
1) Although the longterm safety of AAV based gene therapies is of high importance and broad interest, I am not convinced that this manuscript as presented adds substantial new insights that inform "the longterm efficacy and safety of treating patients with zolgensma." It has already been reported that ICV-delivery of a AAV9-SMN vector driven by the GUSB promoter can result in aggregation of SMN protein in neurons and neurodegeneration (Van Alstyne et al.). It has also been previously demonstrated that AAV vectors at high dose in mice cause HCC in a species-specific way and this locus-specific integration is not relevant to humans. The only novel toxicity is the cardiac thrombosis. Also regarding human relevance, it is not clear that the findings observed with ICV injection of AAV9-AAV are relevant to the current FDA approved zolgensma which is delivered iv. The results of IT delivery of zolgensma have been presented in abstract form: the relative dose of virus used in the human studies is significantly less than the doses being used in humans.

We agree with the reviewer that it was not new that AAV vectors, when administered at high doses by IV injection, lead to HCC in mice. However, this is the first report that an AAV vector for human patients resulted in tumorigenesis in the mouse liver and lung by ICV injection. More importantly, the 2nd-G vector does not raise safety concerns regarding tumor development, underscoring the importance of vector design, especially the promoters in AAV gene therapy.

We also treated the WT mice with the benchmark or 2nd-generation vector by IV injection, which is more efficient than ICV in transducing the mouse liver. Without surprises, liver tumors were observed as early as 6 months in the benchmark vector-treated mice, but not in the 2nd-G vector treatment.

2) Regarding the comparisons of SMA disease efficacy with the different vectors, this manuscript does demonstrate that the improved efficacy with the second-generation vector persists at later time points, but this seems to be an incremental advance compared to their thorough, previously published work.

After changing the administration route from IV in the previous study to ICV in the current study, both vectors increased SMN expression in the CNS and decreased it in peripheral tissues. The changes in

SMN expression patterns toward endogenous SMN profile enable more effective and safer therapy in SMA mice, resulting in less pronounced differences in therapeutic efficacy between these two vectors. However, the 2nd-G vector still shows improvements in animal survival, motor function, and safety profile in SMA mice. SMA patients will be treated by IV or IT, depending on their age. The 2nd-G vector outperforms the benchmark vector in both administration routes, making it a potential candidate for future translational studies.

3) The authors should justify the virus doses used particularly when trying to make conclusions regarding Zolgensma dosing in humans. The doses of zolgensma used in the IT trials in humans are significantly lower than those used in the mice (see STRONG study). The highest dose of virus was only tested in WT mice-was this because it was not tolerated in SMA mice?

The dose at 1.0E+14vg/kg for C57BL/6 WT mice by ICV injection was selected based on the study by Van Alstyne et al (PMID: 33795885, Figure 1). In that study, the same AAV9 dose that expresses SMN under the GUSB promoter was associated with neurodegeneration. We selected a dose of 1.0E+14 vg/kg in WT mice for the benchmark, and the 2nd-generation vector is to assess its safety profile at high doses. A lower dose of Zolgensma by IT may be safe, but therapeutic outcomes may not be ideal. Please see lines 217-218.

During the revision, we injected some SMA mice with 1.0E+14 vg/kg via ICV and found that all treated mice have survived so far (40 days). The 1.0E+14 vg/kg via ICV is tolerated in SMA mice but out of our current research scope.

4) The authors need to define and quantify aggregates of SMN not just show single images. Are these aggregates associated with neurodegeneration as previously reported?

We thank the reviewer for the suggestion. We have co-stained SMN with SmB (SmB is a member of the Sm core that is assembled on small nuclear RNAs by SMN protein), Parvalbumin (PV, is a marker for proprioceptive neurons, PNs) to examine the aggregates in the DRGs. The new data, including quantifications, are shown in Figs. 2d, 4g; Supplementary Fig. 11).

In SMA mice at 12 months of age, we observed neither compromised motor function nor hindlimb clasping behavior from the 2nd-G vector treatment. In WT mice at 20 months, we observed impaired motor function and hindlimb clasping behavior (Fig. 4 b-c), as reported in the previous publication by Van Alstyne et al.

5) There is no histological evidence that the second-generation vector outperforms the benchmark. MN numbers are equivalent among experimental groups. Electrophysiology, NMJ or myofiber pathological assessment would help provide convincing evidence that one vector outperforms another in terms of the most clinical relevant cell types: motor neurons and myofibers.

We have conducted the following experiments as the reviewer suggested:

1. We quantified the ChAT(+) neurons in the L1 and L5 segments and found that the 2nd-G vectors outperform BMK in rescuing motor neurons. The new data are shown as Figs. 1h, 2e; Supplementary Figs.1, 5, 10.
2. We also conducted electrophysiological testing on SMA mice that had received AAV treatment for 12 months. The compound muscle action potential (CMAP) amplitude in the 2nd-G vector-treated SMA mice is comparable with HC controls, but not the BMK vector. The new data is shown as Fig. 2g.
3. In addition, we examined the NMJs of the quadratus lumborum (QL) muscles, which are vulnerable in the SMA mice. We observed that both vectors can restore NMJ innervation, but the

2nd-generation vector is more effective than the BMK vector. The analysis was done in a double-blind manner. The new data is shown as Fig. 1j.

6) The authors should be more upfront about the hepatocellular carcinoma being species specific in the abstract. Although discussed in the discussion, it needs to be mentioned earlier.

Revised as lines 73-76; 97-99

7) The authors make reference to results of the IT dosing Zolgensma clinical trials. The authors should review the abstracts reporting these results from the World Muscle Society meeting and Cure SMA meetings that have been posted on the Novartis website. The efficacy is extremely modest. This should be more accurately reported in the introduction.

Revised as lines 66-67

Responses to reviewers

REVIEWER COMMENTS

Reviewer #1 (Remarks to the Author):

The authors have performed additional experiments and revised their manuscript accordingly.
I have no new concerns.

Response: We thank the reviewer for the constructive and detailed comments, which have greatly improved the manuscript.

Reviewer #2 (Remarks to the Author):

it would have been helpful to get a more detailed reply than 'see line XX' but I think I went through everything and with the reponse to the other reviewers belief all important points have been addressed sufficiently

Response: We apologize for any inconvenience caused by our earlier responses and greatly appreciate the reviewer's careful reading and helpful suggestions.

Reviewer #3 (Remarks to the Author):

The authors have done a very good job of addressing the concerns that were raised during the initial review. They have edited the manuscript to make clear that HCC may be a rodent specific phenomena. In addition, the authors have performed new experiments to quantify motor neuron number and size, NMJ innervation and determine CMAPs. In addition SMN aggregates have been quantified in DRGs.

I have a few remaining concerns:
1) For this reviewer, normalizing motor neuron number and size values to 100% is not appropriate and makes it very difficult to judge the validity of these assessments. I strongly recommend showing absolute values for MN number and MN size. In addition for most of these analyses, there do not appear to be statistically significant differences observed between the benchmark vector and the 2ndGen vector. Given this, these results should not be overstated (a lack of difference with WT and 2nd gen mice is less convincing than a statistically significant improvement between 2nd generation vs. benchmark vector-treated mice).

Response: This is a fair point. Presenting the data as a percentage of the control makes it hard to see the actual numbers, so we agree that showing the absolute values is much clearer. In the revised manuscript, we now show the absolute values for MN number and soma size in both the main figures and the supplementary material (revised Figs. 1h, 2e, 4e, Supplementary Figs. 1, 5, and 11). As the reviewer notes, differences between the benchmark and 2nd-generation vectors are not statistically significant in most cases. We now explicitly label the p-values for comparisons between these two groups in the figures for clarity, and we have tempered our interpretation accordingly in the Results and Discussion sections (Lines 170–172).

2) Both reviewer 1 (point#9) and reviewer 3 (point #3) raised concerns about claims that 2nd gen vector is safer, BUT WT mice treated with a high dose showed more motor behavioral abnormalites than bench mark vector (figure 4). The authors speculate that this may be due to presence of mouse Smn in addition to virally-delivered SMN. They note in response letter that during the revision period they treated SMA mice with high dose-I think the longterm results of the effects of the bench mark vs. 2nd gen high dose vector in the SMA mice would be ideal to include.

Response: We appreciate the reviewer's careful attention to our safety claims. We acknowledge that, at high doses in WT mice, the 2nd-generation vector was associated with more pronounced motor abnormalities than the benchmark vector. We have now included a 90-day follow-up study in SMA mice treated with high-dose AAV, including both the benchmark and 2nd-generation vectors (Lines 182–188 and Supplementary Fig. 8). SMA mice were injected intracerebroventricularly at P0 with 1.0 × 10^14 vg/kg. Over the 90-day period, all animals in the 2nd-generation vector group survived (n = 7), whereas one animal in the benchmark group (n = 7) died (Supplementary Fig. 8a). There were no significant differences in body weight gain or rotarod performance between the two treatment groups. Notably, however, 2nd-generation vector–treated SMA mice, but not benchmark vector–treated mice, showed rotarod performance comparable to WT mice on day 90 (Supplementary Fig. 8b–c). In light of these findings, we have revised the manuscript to present the safety profile of the 2nd-generation vector more cautiously and to emphasize its potential for CNS and DRG-related neurological toxicity (Lines 348–349).

3) The publications reporting the experience with IT zolgensma in humans have now been published (PMID: 41360995 and PMID: 41360993). These manuscripts need to be cited and appropriately discussed. I remain concerned about the wording of the last phrase of the abstract: "...these findings raise concerns regarding the long-term safety and efficacy of Zolgensma in SMA patients." Given the many differences between mice and humans (anatomy, CSF volume, blood brain barrier, immunology, etc), I think this conclusion requires softening. On this theme, a point that has not been sufficiently addressed in the discussion is the relative doses of virus per CSF volume administered in mice in this study vs. human clinical use. In humans, 1.1x10(14)vg is given IT with a human CSF volume estimated at 100-150 ml. In contrast, mouse CSF volume is ~35-50 ul. I think some discussion of the estimated concentrations of virus in CSF is important to include particularly as level of SMN overexpression seems to be a key determinant of toxicity.

Response: We thank the reviewer for drawing our attention to the recently published IT Zolgensma data and for prompting a more nuanced discussion of CSF dosing and the wording of our conclusions. We have now cited these two studies and discussed their translational relevance in the manuscript. We have also revised the final sentence of the abstract to: "these findings underscore the need for careful long-term monitoring in patients treated with Zolgensma," to better reflect the limitations of extrapolating from mouse to human.

In these two trials (NCT05386680 and NCT05089656), a total of 1.1E+14 vector genomes were injected into the CSF of human patients weighing around 10-35 kg. In line with the reviewer's point about vector genome/CSF volume, we now explicitly discuss the relative CSF concentrations. The estimated CSF volume in humans and neonatal mice is 100-150 milliliters and 3-8 microliters, respectively. Therefore, the CSF vector concentration in our mouse experiments is 8.5-68-fold higher than in clinical conditions (0.6-5.0E+13 vg/mL in mice vs 0.7-1.1E+12 vg/mL in humans). Compared to the vector dose in our mouse studies based on body weight (3.3E+13 vg/kg in the SMA mice and 1.0E+14 vg/kg in WT mice), the SMA patients were treated at 9-32-fold lower doses (3.1E+12 to 1.1E+13 vg/kg in humans). The high vector dose in the mouse studies may overestimate the safety concerns of the benchmark vector, including the HCC risk. We have incorporated this discussion into the revised Discussion section (Lines 401–412).
